# Cu(II)-Catalysed Hydrocarboxylation of Imines Utilizing CO_2_ to Synthesize α-Unsaturated Aminocarboxylic Acids

**DOI:** 10.3390/ph15101240

**Published:** 2022-10-09

**Authors:** Allen T. Gordon, Eric C. Hosten, Adeniyi S. Ogunlaja

**Affiliations:** Department of Chemistry, Nelson Mandela University, Gqeberha P.O. Box 77000, South Africa

**Keywords:** Schiff bases, CO_2_, α-unsaturated carboxylic acids, hydrocarboxylation, Cu(II) catalysts

## Abstract

Here, we report the Cu(II)-photocatalysed hydrocarboxylation of imines (C=N) from a series of synthesized Schiff Base derivatives, namely *(E)-1-(4-((4-methylbenzylidene)amino)phenyl)ethanone*, (*E*)-1-(3-((5-bromo-2-hydroxybenzylidene)amino)phenyl)ethanone, (E)-4-((5-bromo-2-hydroxybenzylidene)amino)-1,5-dimethyl-2-phenyl-1H-pyrazol-3(2H)-one, and (E)-1,5-dimethyl-4-((4-methylbenzylidene)amino)-2-phenyl-1H-pyrazol-3(2H)-one, with carbon dioxide (CO_2_) to generate disubstituted amino acids. Under mild conditions (atmospheric pressure of CO_2_, room temperature, and 30 W Blue LED light), good to excellent yields confirming the formation of substituted amino acid unsaturated acid derivatives were obtained. Single crystal X-ray diffraction (SC-XRD) and UV-Vis diffuse reflectance spectroscopy (UV-Vis-DRS) confirmed the square pyramidal geometry of the Cu(II) photocatalyst. Docking and DFT calculations of the substituted amino acid unsaturated acid derivatives showed their potential as antimicrobial molecules.

## 1. Introduction

Carbon dioxide (CO_2_) is a greenhouse gas that is found in the atmosphere, and human activities, such as energy generation by fossil fuel combustion, dominate CO_2_ emissions. The release of large amounts of CO_2_ into the atmosphere poses an environmental problem that must be addressed, paving a way for investigating routes that can allow for the use of CO_2_. A route that deals with excess CO_2_ involves using it as a carbon synthon in organic synthesis. Given carbon dioxide’s high kinetic and thermodynamic stability [1], activating it using photoredox catalysts has enabled CO_2_ conversion via incorporation into organic compounds [2,3]. In recent decades, transition-metal-catalysed carboxylation using CO_2_ has received considerable attention. To take inspiration from natural photosynthesis, this study will focus on devising reaction routes for the fixation of CO_2_ with organic substrates in the synthesis of α-amino acids via the use of photocatalysts. Ir(III)-based complexes have been employed as choice photocatalysts owing to their strong absorption, long lifetimes of their excited states, and high redox potentials. For example, [Ir(ppy)2(4,4′-tBu2-bpy)]PF_6_ (catalyst) efficiently promoted the hydrocarboxylation of an imine with CO_2_, producing the desired product with an excellent yield [3]. Nonetheless, these photocatalysts are expensive, which limits their applications [4]. In the search for an alternative metal-based catalyst, copper has emerged as an attractive complement due to its redox properties [4]. Microbial resistance against current drugs is a topic of serious concern and is predicted to worsen in the coming years [5]. Considering the predicted increase in antibiotic resistance, the development of new potent compounds with antibacterial activity is vital. The introduction of amino acids to Schiff Bases via copper-catalysed hydrocarboxylation may offer improved medicinal activity [6,7,8,9]. Furthermore, Schiff Bases and α-unsaturated carboxylic acids are significant in the design of pharmaceuticals, since they possess an array of biological properties [6,7,8]. Carboxylic acid (-COOH)-containing drugs play a major role in the medical treatment of pain and diseases [8]. In this study, we set out to hydrocarboxylate synthesised Schiff Base derivatives, namely (E)-1-(4-((4-methylbenzylidene)amino)phenyl)ethenone (**1**), (E)-1-(3-((5-bromo-2-hydroxybenzylidene)amino)phenyl)ethenone (**2**), (E)-4-((5-bromo-2-hydroxybenzylidene)amino)-1,5-dimethyl-2-phenyl-1H-pyrazol-3(2H)-one (**3**), and (E)-1,5-dimethyl-4-((4-methylbenzylidene)amino)-2-phenyl-1H-pyrazol-3(2H)-one (**4**), with carbon dioxide (CO_2_) to produce α-substituted amino acids under blue light irradiation by using [Cu(phen)_2_Cl]. After the hydrocarboxylation of Schiff Bases, we achieved the highest conversion rate of 60% of the substituted amino acid yield when using [Cu(phen)_2_Cl] (100.57 mg) as a photocatalyst, CyNMe_2_ (2 mL), and MeCN (10 mL). It is worth mentioning that, when the photocatalyst was not present, less than 5% carboxylation occurred. The presence of COOH in the synthesised Schiff bases (α-substituted amino acids) influenced the interaction with E. faecalis (4M7U) and DNA through hydrogen bonding and mixed π-interactions.

## 2. Results and Discussion

### 2.1. Photocatalyst Characterization

#### 2.1.1. UV-Vis and Band Gap

The UV portion of [Cu(phen)_2_Cl] was characterized by the intense ligand-centred (LC) bands typical of the π–π* transitions of phenanthroline ligands between 250 and 300 nm and a metal-to-ligand charge transfer (MLCT) near 400 nm. The d–d transition, observed around 670 nm, suggested the formation of a pentacoordinate copper(II) centre in a square pyramidal geometry (Figure S1a). The DFT-simulated UV-Vis data also presented strong absorption bands in the 329–370 nm range (prominent in theoretical UV-Vis, Figure S1b) typical of square-pyramidal geometry. Figure S2 shows that (αthv)^0.5^ versus hv had regions with linear fitting. This indicates the indirectly allowed optical transitions in the ligand and its complexes [9,10,11,12]. The extrapolation to the hv axis from the graph of (αthv)^0.5^ versus h*v* presented band gaps of 0.54, 0.80, and 1.99 eV, thus confirming its high electron transfer properties (Figure S2). 

#### 2.1.2. Crystal Structure: [Cu(phen)_2_Cl]

**Figure 1:**
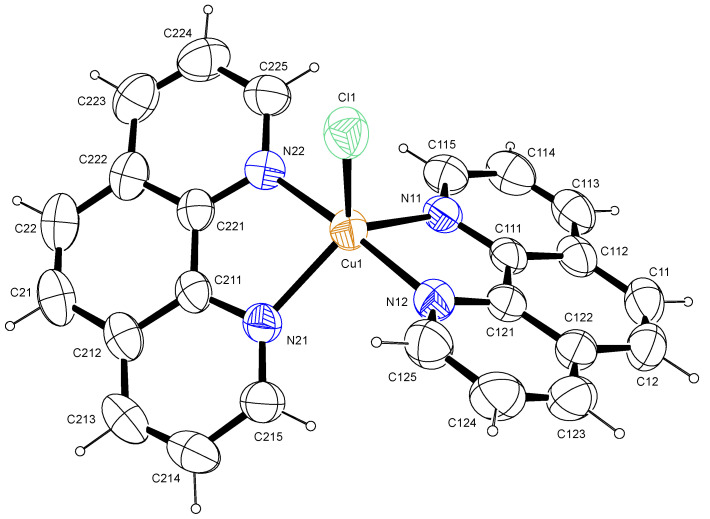
Labelled diagram of [Cu(phen)_2_Cl]. Ellipsoids were drawn at 50% probability level.

In the title complex, similar to reported data [13], the copper atom was five-coordinated by four N atoms from two 1,10-phenanthroline ligands and one Cl atom attached at the apical region, as shown in the ORTEP view (Figure 1). This complex crystallized in a monoclinic crystal system and in a C2/c (No.15) space group (Table 1). The structure had a number of large, interlinked voids (total void volume 1732 Å^3^, 33 % of the unit cell volume) in which the solvent and/or counter ions were extensively disordered. Owing to this extensive disorder, the nature of the solvent and/or counter ions could not be determined with certainty. Platon’s squeeze routine was, therefore, used to remove the void residual electron density. The residual electron density in the void was calculated to be 560 electrons per unit cell, which equated to 70 electrons per asymmetric unit cell. A Cu(II) complex was suggested by the NMR studies, which showed that the compound was paramagnetic. Ethanol was used to prepare the crystals and, assuming that the counter ion was chloride, suggested that the formula was [Cu(II)(phen)_2_Cl]Cl.2EtOH (C_28_H_28_Cl_2_CuN_4_O_2_, 587.01 g/mol). The Cu–N bond lengths were in the range of 1.9769(17)–2.1073(19) Å and the Cu–Cl bond distance was 2.3368(7) Å (Table 2), which is consistent with the results of previous studies [13,14]. The coordination geometry around the central Cu atom was a distorted trigonal bipyramid with N11, N21 and Cl1 as the trigonal base and N12 and N22 as the apex atoms. Which is consistent with the observed τ value of 0.13 [15]. However, the cis bond angles of the Cu atom with nitrogen ranged from 81.30(12)°–95.01(12)°, while the trans angles ranged from 123.06(11)–175.88(13)°, further corroborating the distorted trigonal bipyramid geometry.

**Figure 2:**
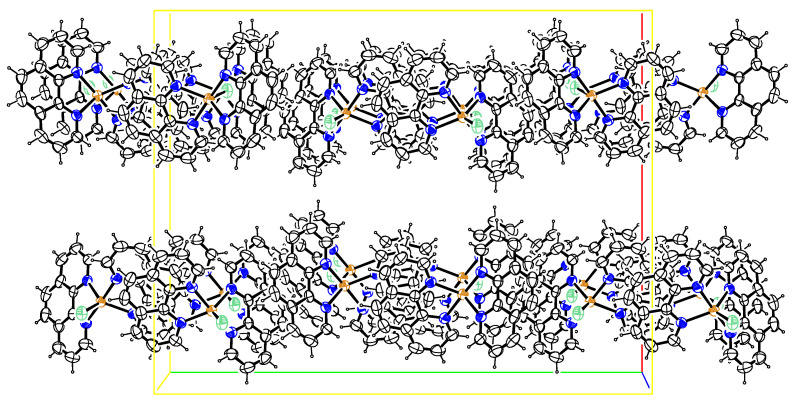
Crystal packing diagram drawn normal to (001). Ellipsoids are drawn at the 50% probability level for the photocatalyst.

The crystal packing diagram for ([Cu(phen)_2_Cl] is depicted in Figure 2. There were extensive intermolecular π…π ring interactions between the phenanthroline ligands with centroid-to-centroid distances varying from 3.5493(13) to 3.8073(12) Å (Table 3). These interactions, together with the intermolecular Cu1―Cl1…π ring and C21―H21…Cl1 interactions of 3.5854(10) and 2.76 Å, respectively, linked the complexes together in planes parallel to the (1 0 0) plane. These intra- and inter-molecular interactions were effective in stabilizing the crystal structure of these complexes and the formation of the 3D supramolecular assemblies [16,17,18].

### 2.2. Schiff Bases and Corresponding Amino Acid (Hydrocarboxylation)

#### 2.2.1. Schiff Bases 

The IR of the synthesized Schiff bases confirms the formation of an imines band v(C=N) at 1640–1530 cm^−1^ [19], and C–H bands were observed at characteristic vibrations of 1450–1400, 1100–1090, and 760–720 cm^−1^ [20]. The absence of bands characteristic of primary amine ν(NH) confirms the formation of the imine moiety. Furthermore, the ^1^H NMR spectra confirm the proposed stoichiometry of the envisaged Schiff bases. The characteristic C=N–H signal in the ^1^H NMR appeared at 8.36 ppm for 2. The proton signals of compounds 3 and 4 for the C=N-H group were de-shielded, appearing at 9.56 and 9.76 ppm, respectively. The integration of the protons in each corresponding compound’s ^1^H NMR and respective coupling constants evidenced that the desired compounds were afforded. Aromatic protons peaked as singlets, doublets, and multiplets. The information obtained from ^13^C shows the disappearance of the aldehydic carbon (185–210 ppm) in the spectra of the Schiff bases. The overall number of carbons in the spectra for the synthesized Schiff bases was consistent with that desired for each Schiff base. DFT studies were conducted to understand the frontier molecular orbitals of the synthesized Schiff bases. The ^1^H NMR, ^13^C NMR, and FT-IR of (I-1-(4-((4-methylbenzylidene)amino)phenyl)ethenone (Figure S4), (E)-3-((4-(dimethylamino)benzylidene)amino)phenol (Figure S5), (E)-4-((4-hydroxybenzylidene)amino)-1,5-dimethyl-2-phenyl-1H-pyrazol-3(2H)-one (Figure S6), and aI(E)-1,5-dimethyl-4-((4-methylbenzylidene)amino)-2-phenyl-1H-pyrazol-3(2H)-one (Figure S7) can be found in the Supplementary Data section.

#### 2.2.2. Hydrocarboxylation Reaction

The Schiff base ((E)-1-(4-((4-methylbenzylidene)amino)phenyl)ethenone (1) with a methyl group yielded 58% of 2-(4-(dimethylamino)phenyl)-2-((3-hydroxyphenyl)amino)acetic acid (5) after hydrocarboxylation with CO_2_. The substitution of the methyl group for dimethylamino, an electron-donating group, produced 60.1% of 2-(4-(dimethylamino)phenyl)-2-((3-hydroxyphenyl)amino)acetic acid (6) (Table 4). 

Pyrazol-based molecules with methyl (Me) and hydroxyl (OH) groups were also examined. The hydrocarboxylation of the molecule with Me into the N-phenyl ring molecule (3) reacted to provide 45.7% of 2-((1,5-dimethyl-3-oxo-2-phenyl-2,3-dihydro-1H-pyrazol-4-yl)amino)-2-(4-hydroxyphenyl)acetic acid (7) after 24 h. The reaction of the pyrazol-based molecule with OH–Ph (4) produced the hydrocarboxylated product 2-((1,5-dimethyl-3-oxo-2-phenyl-2,3-dihydro-1H-pyrazol-4-yl)amino)-2-(p-tolyl)acetic acid (8) with a yield of 50.7%, thus indicating that the electron-donating properties of the OH group may have influenced the increased yield under similar reaction conditions (Table 4). Generally, where yields were lower, by-products derived from the imine reduction were observed (^1^H and ^13^C NMR data of the hydrocarboxylated products are provided in the Supplementary Data section, Figures S4–S11). 

#### 2.2.3. Mechanistic Pathway of Cu(II) Photocatalyst

**Figure 3:**
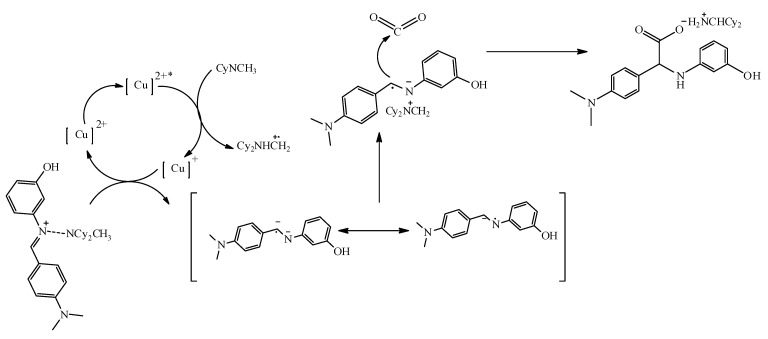
Plausible mechanism of Cu(II) photoredox catalysis.

A simple UV-Vis experiment was conducted to explore the mechanism of the Cu(II) catalyst. The UV–vis spectra indicated that the Cu^2+^ catalyst was able to absorb visible light (UV-Vis absorption study showed that, before irradiation, there was a peak present at ~450 nm (metal-to-ligand charge transfer) and ~740 nm (d–d transition)); hence, the hydrocarboxylation reaction was likely initiated by the irradiation of Cu^2+^ by light to produce its excited state, Cu^2+^*. The introduction of a base and BIH in the presence of LED light resulted in a decrease in the intensity of the peak between 650 and 800 nm, thus confirming the disappearance of d–d transitions due to electron transfer from the base to the metal centre (Cu(II)), giving rise to Cu(I) that did not contain the d–d transitions found in Cu(II). The disappearance of the d–d transition region, therefore, confirmed the conversion of Cu(II) to Cu(I) through electron donation (Figure S3). Below is the proposed reaction pathway for the synthesis of hydrocarboxylated compounds **5**–**8** (Figure 3) [3].

#### 2.2.4. FT-IR

1,10-Phenanthroline exhibited absorption bands in the regions of 1519–1643 ṽ (C=N) and 2095–3091 ṽ (C-H). While the corresponding copper(II) complex (Cat) showed absorption bands at 3355 ṽ(OH), 3068 ṽ (CH), 2981 ṽ(CH), 2344 ṽ(CH), 1602 ṽ(C=N), 1428 ṽ(C=N), and 424 ṽ(Cu–N) (Figure S3). The presence of ṽ(OH) confirmed the presence of water in the coordination sphere of the complex. Numerous weak absorption bands were observed in the range of 3080–2350 cm^−1^ and were assigned to the C–H stretching of 1,10-phenanthroline rings. The observed C=N band shift in the complexes was due to the coordination of the nitrogen ligands to copper(II) atoms [21,22]. The complexes presented a ṽCu–N band between 424 and 414 cm^−1^. 

### 2.3. Theoretical Studies 

#### 2.3.1. Chemical Descriptors of [Cu(phen)_2_Cl]

**Figure 4:**
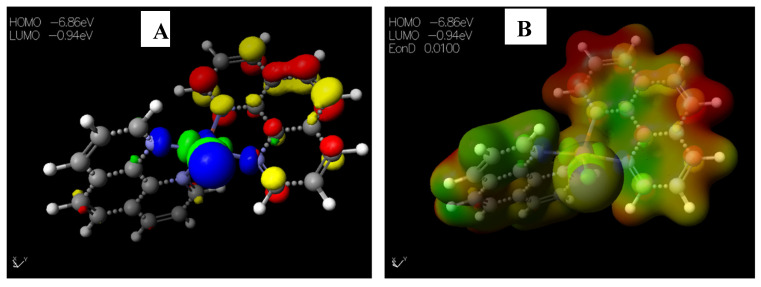
Images showing the (**A**) Highest occupied molecular orbital (HOMO) and Lowest unoccupied molecular orbital (LUMO) and (**B**) Molecular electrostatic potential (MESP) plots of [Cu(phen)_2_Cl].

The HOMO−LUMO gap, which describes electric transport properties [23,24,25], showed that the HOMO energies originated from the aromatic rings of [Cu(phen)_2_Cl], while the LUMO energies originated from the copper and chlorine atoms coordinating to the phenanthroline rings (Figure 4A). The MESP map of the complex (Figure 4B) showed the two 1,10-phenanthrolines in a greenish-yellow colour, thus representing a slight electron-rich-a zero potential [26].

#### 2.3.2. Chemical Descriptors of Schiff Bases and Envisaged Unnatural α-amino Acids

**Figure 5:**
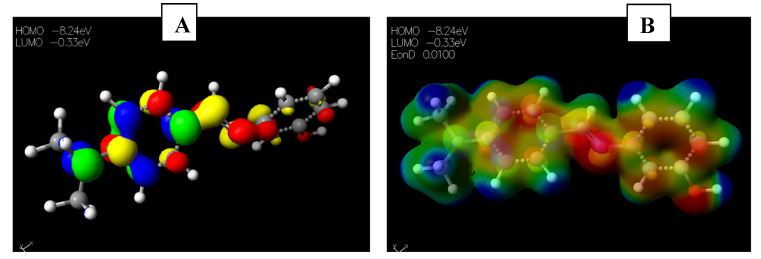
Images showing the (**A**) Highest occupied molecular orbital (HOMO) and Lowest unoccupied molecular orbital (LUMO) and (**B**) Molecular electrostatic potential (MESP) plots of **1**.

**Figure 6:**
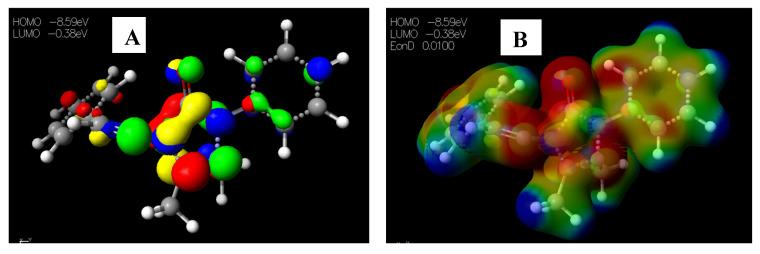
Images showing the (**A**) Highest occupied molecular orbital (HOMO) and Lowest unoccupied molecular orbital (LUMO) and (**B**) Molecular electrostatic potential (MESP) plots of **2**.

**Figure 7:**
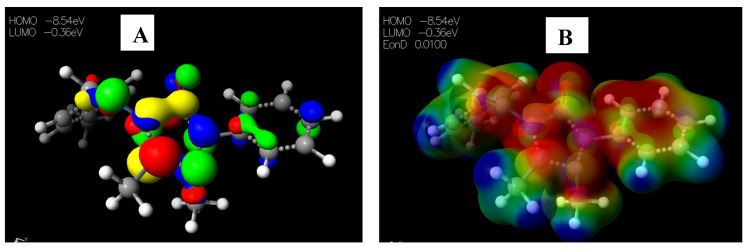
Images showing the (**A**) Highest occupied molecular orbital (HOMO) and Lowest unoccupied molecular orbital (LUMO) and (**B**) Molecular electrostatic potential (MESP) plots of **3**.

**Figure 8:**
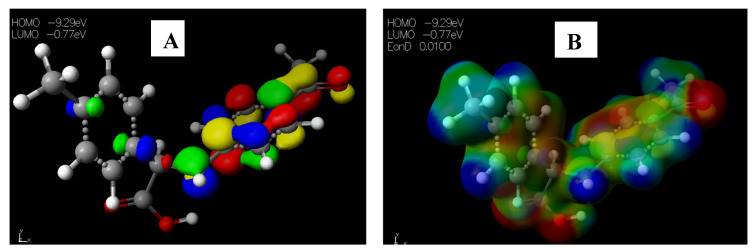
Images showing the (**A**) Highest occupied molecular orbital (HOMO) and Lowest unoccupied molecular orbital (LUMO) and (**B**) Molecular electrostatic potential (MESP) plots of **4**.

**Figure 9:**
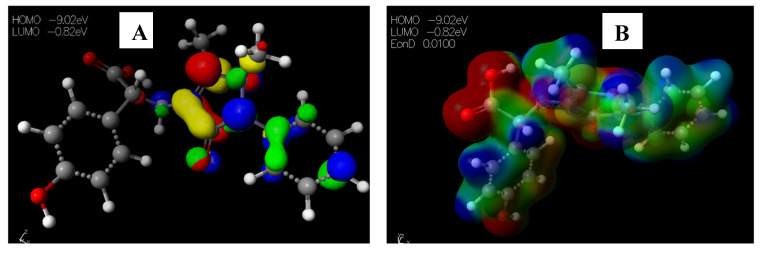
Images showing the (**A**) Highest occupied molecular orbital (HOMO) and Lowest unoccupied molecular orbital (LUMO) and (**B**) Molecular electrostatic potential (MESP) plots of **5**.

**Figure 10:**
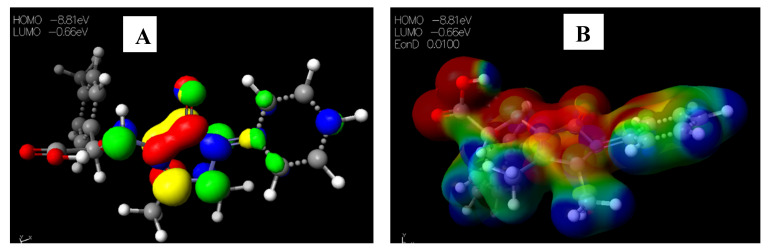
Images showing the (**A**) Highest occupied molecular orbital (HOMO) and Lowest unoccupied molecular orbital (LUMO) and (**B**) Molecular electrostatic potential (MESP) plots of **6**.

**Figure 11:**
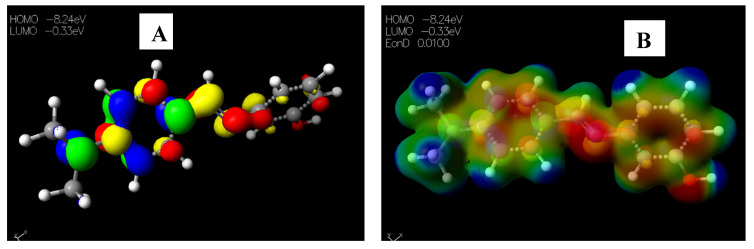
Images showing the (**A**) Highest occupied molecular orbital (HOMO) and Lowest unoccupied molecular orbital (LUMO) and (**B**) Molecular electrostatic potential (MESP) plots of **7**.

**Figure 12:**
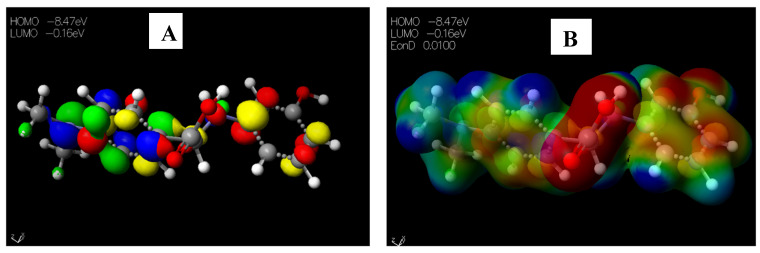
Images showing the (**A)** Highest occupied molecular orbital (HOMO) and Lowest unoccupied molecular orbital (LUMO) and (**B**) Molecular electrostatic potential (MESP) plots of **8**.

Schiff base **2** and hydrocarboxylated product **6** presented the highest E_HOMO_ = −8.25 and −8.47 eV, while compound **7** (Table 5) has the highest E_LUMO_ =−0.16 eV. Therefore, **2** and **6** can donate electrons to an electron-poor species, while **7** has the propensity to accept electrons. The MESP plots show that the aromatic rings were of neutral potential, and the functional groups COOH and HC=N had a red surface corresponding to electron-rich groups. The hydrogens on NCH_3_ had a blue surface and thus represented regions that were electron-deficient. The images depicting the HOMO–LUMO (**A**) and MESP mapping (**B**) of compounds **1**–**8** is provided in Figure 5, Figure 6, Figure 7, Figure 8, Figure 9, Figure 10, Figure 11 and Figure 12.

#### 2.3.3. Docking Studies

Molecular docking is a computational technique that predicts the relative orientation of one molecule when bound with an active site of another molecule to form a stable complex, such that the free energy of the overall system is minimized [27]. In this study, molecular docking was conducted to determine the mode of the interaction with enzyme E. faecalis DHFR (4M7U) and relative orientation of the B-DNA dodecamer (1BNA) with synthesized compounds **1**–**8** and antibiotics (trimethoprim and ciprofloxacin) as standard drugs [28,29]. The standard drugs were chosen on the basis that they bore some similar functional groups to those of the synthesized compounds. Ciprofloxacin bore CO_2_H functionality, which was also present in **5**–**8**, and trimethoprim bore the imine (C=N) group, which was also present in Schiff bases **1**–**4**. Furthermore, these standard drugs exhibited greater bioavailability, higher plasma concentrations, and increased tissue penetration, as reflected in their greater volume of distribution [28].

G-score was used as the typical measure of the docking results. A more negative G-score represented the best-docked compounds, i.e., better binding affinity. The glide module of Schrödinger suite 2022-1 was used to dock synthesized compounds **1**–**8** and the two standard drugs into the active site of E. faecalis DHFR (4M7U), downloaded from the protein data bank (https://www.rcsb.org/structure/4M7U, accessed on 6 September 2022), with a resolution of 2.10 Å. The docking results showed that the synthesized compounds, particularly α-unsaturated amino carboxylic acids **5**–**8**, all exhibited a more negative G-score than the two standard drugs. Furthermore, **5** was the best-docked compound, evidenced by the G-score of −5.93 kcal/mol, E-model of −59.663 kcal/mol, and ligand efficiency of −0.281 kcal/mol (Table 6). Schiff bases **1**–**3** displayed more negative G-scores than that exhibited by ciprofloxacin (G-score = −4.228 kcal/mol), yet lower scores than that of trimethoprim (G-score = −5.837 kcal/mol). The least-docked compound to the enzyme 4M7U was observed on Schiff base **4**, with a G-score of −3.255 kcal/mol, E-model of −29.960 kcal/mol, and ligand efficiency of −0.140 kcal/mol (Table 6). Prime MM-GBSA calculations were carried out using the Glide pose viewer files generated from the glide calculations to estimate the relative binding affinity of the ligands (synthesized compounds and standard drugs) to the receptor 4M7U, and the results are reported in kcal/mol. As such, the MM/GBSA binding energies were the estimated free energies of binding, and a more negative value indicates stronger binding [30]. Along with the binding free energies (ΔG Bind), other values estimated include the electrostatics interaction energy or coulomb (ΔG Coulomb), Van der Waals interaction energy (ΔG vdW), and generalized Born electrostatic solvation energy ΔG Solv_GB, among others (Table 7). As such, synthesized α-unsaturated amino carboxylic acid **7** with a binding free energy of −41.45 kcal/mol exhibited the best binding affinity, while Schiff base **2** had a binding free energy of −29.23 kcal/mol. Compound **8** exhibited the highest Van der Waals interaction energy (ΔG vdW = −37.97 kcal/mol), while the standard drug Ciprofloxacin exhibited the highest generalized Born electrostatic solvation energy (ΔG Solv_GB = 50.27 kcal/mol). 

**Figure 13:**
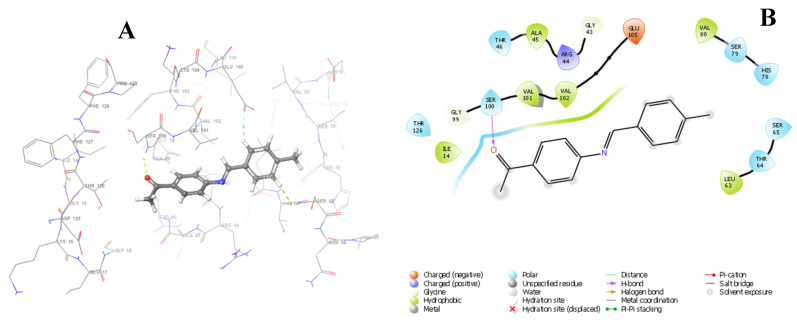
Three-dimensional binding model of **1** with 4M7U (**A**); two-dimensional diagram of **1** and interacting residues (**B**).

The docking results of Schiff base **1** into the active site of E. faecalis DHFR (4M7U) revealed the involvement of the hydrogen bonding interaction between 4M7U and amino acid residue SER_100_ (1.86 Å) (Figure 13). Schiff base **2** exhibited π–cation interactions with the amino acid residue ARG_44_ (6.26 Å) and strong hydrogen bond interactions with amino acid residue SER_65_ (1.80 Å) (Figure S12). A hydrogen bond interaction between **3** and residues ALA_45_ (2.69 Å) and SER_65_ (2.05 Å) was observed. Schiff base **4** interacted with residues of 4M7U via hydrogen bonding with VAL_101_ (2.73 Å) and GLY_99_ (2.68 Å). Synthesized α-unsaturated amino carboxylic acids **5**–**8** interacted with the protein 4M7U with more than one hydrogen bond. For instance, the oxygen atom on **5** interacted with residues VAL_102_ (2.04 Å), ARG_44_ (2.60 Å), SER_65_ (1.88 Å), and THR_64_ (2.07 Å). Unfavourable π-alkyl interactions between **5** and residue SER_65_ with bond lengths of 2.38 and 2.29 Å were also displayed. Hydrogen bonding occurred between compound **6** and residues GLU_105_ (1.75 Å) and SER_65_ (2.63 Å) (Figure S16). There was a weak interaction between **7** and amino acid residue ARG_44_ corresponding to π-cation interaction. Carboxylic acid **7** also interacted with amino acids via hydrogen bonding through THR_126_ (1.90 Å), ALA_45_ (1.80 Å), THR_46_ (2.60), and GLY_99_ (2.60 Å). Carboxylic acid **8** also interacted with the binding residues via hydrogen bonding with residues ALA_45_ (1.83 Å) and THR_46_ (2.60 Å). The hydrogen bonds and mixed π-interactions of Schiff bases (**1**–**4**) and those of α-unsaturated amino carboxylic acids (**4**–**8**) with Enterococcus faecalis enzyme (4M7U) are presented in Table 8. The 3D and 2D interaction images of standard drugs and compounds **2**–**8** with 4M7U are provided in the Supplementary Data.

**Figure 14:**
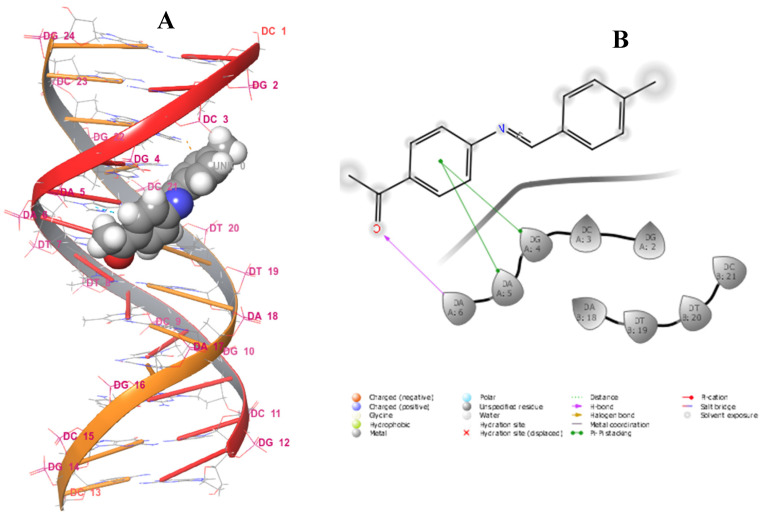
Three-dimensional binding model of **2** with 1BNA (**A**); two-dimensional diagram of **2** and interacting residues (**B)**.

The sequence of DNA base pairs defines the characteristics of individuals, ranging from physical traits to disease susceptibility [31]. Notably, the double helix of DNA is of great length and short diameter, consisting of minor and major groove regions. The minor groove is narrow and shallow, only about 10 Å in width. The major groove is deeper and wider, approximately 24 Å in width [32]. It has been reported in the literature that small organic and inorganic molecules bind to DNA and influence several biological processes, such as transcription and replication. These molecules can modify, inhibit, or activate the functioning of DNA, acting as therapeutics for the treatment and prevention of diseases. Other studies have also characterized a variety of molecules that interact with DNA, which are classified as antibiotic, antitumor, antiviral, or antiprotozoal agents. Hence, it is important to investigate the interaction between drugs or drug prototypes and DNA to understand their mechanism of action at the molecular level [33,34,35,36,37]. Hence, the synthesized compounds **1**–**8** and standard drugs were docked to DNA dodecamer (1BNA) obtained from protein data (https://www.rcsb.org/structure/1BNA, accessed on 6 September 2022) to determine the relative orientation of the docked compounds in the DNA. The evaluation of the docking data with DNA reflected that all of the compounds formed bonds in the minor groove, including ciprofloxacin. Trimethoprim was observed to bind in the major groove (Figure S28). The structures of ciprofloxacin and trimethoprim are presented in Figure S30. Schiff base **2**, with a G-score of −5.815 kcal/mol, represented the best-docked compound and was surrounded by several nucleotides, namely DA_18_, DT_19_, DT_20_, DC_3_, DG_2_, DG_4_, DA_5_, and DA_6_, in the major groove of DNA (Figure 14). Furthermore, Schiff base **2** exhibited hydrogen bonding with nucleotides DA_18_ (2.37 Å) and DC_3_ (5.21 Å) Table 8. The least-docked compound **7** had a G-score of −2.369 kcal/mol, E-model of −62.981 kcal/mol, and ligand efficiency of −0.130 kcal/mol, and exhibited hydrogen bonding with the nucleotides DC_3_ (1.64 Å) and DC_21_ (2.74 Å). Ciprofloxacin, with a G−score of −3.369 kcal/mol, only showed better binding affinity than that of α-unsaturated amino carboxylic acids **5**, **7**, and **8**. Only two compounds (**2** and **4**) exhibited better binding affinity than Trimethoprim (G-score of −4.289 kcal/mol). MM−GBSA binding free energy analysis was carried out on the synthesized compounds and standard drugs to assess the affinity of the ligands to DNA dodecamer (1BNA), and the contributing factors calculated are mentioned in Table S1. Among all of the studied complexes, the ciprofloxacin and DNA complex showed the highest binding free energy (ΔG Bind = −36.19 kcal/mol), while α-unsaturated amino carboxylic acid **8** displayed the lowest binding free energies (ΔG Bind = 0.23 kcal/mol). Schiff base **2**, however, displayed the highest binding free energy among all of the synthesized compounds, even higher than that of trimethoprim. Schiff base **2** also exhibited the highest lipophilic interaction energy (ΔG_lipophilic_ = −8.24 kcal/mol). All compounds displayed a value of 0.00 kcal/mol for hydrogen bonding correction (ΔG_Hbond_ and π-π packing correction (ΔG_Packing_). 

#### 2.3.4. ADMET Properties

All the screened compounds agreed with Lipinski’s rule of five, and only one compound, α-unsaturated amino carboxylic acid **7**, exhibited one violation of Jorgensen’s rule of three. The pharmacokinetic ADME properties play a significant role in the determination of the safety and efficacy of drug-like compounds. Thus, properties, such as the molecular weight (MW), polar surface area (PSA), and Caco-2 cell permeability in nm/s (QPPCaco), were evaluated and compared with the values obtained for the reference drugs, trimethoprim and ciprofloxacin (Table 9). Molecules violating more than one of these rules could have a problem in terms of their bioavailability [38]. Furthermore, all other quantities were within the recommended limit for drug-like properties (Table S2).

## 3. Materials and Methods 

### 3.1. General Chemistry Methods 

A Vario EL Cube Elemental Analyzer was used for the CHNS analysis of the Schiff bases. A Bruker Tensor 27 platinum ATR-FTIR spectrometer was used to record the infrared spectra of molecules in the wavelength range of 4000–400 cm^−1^. UV-Vis was recorded on a Shimadzu UV-VIS-NIR Spectrophotometer UV-3100 (solid reflectance) within a wavelength range of 200–800 nm; the Energy gap E_g_ of [Cu(phen)_2_Cl] was also determined from the UV-Vis data. A Bruker APEX II CCD diffractometer with graphite-monochromated Mo Kα radiation at 298 K with APEX2 [39] software was used for data collection. The cell refinement and data reduction were carried out using SAINT [39]. Crystal data were corrected for absorption effects using the numerical method implemented in SADABS [39]. SHELXT-2018/2 [40] and SHELXL-2018/3 were used to solve the structures by dual-space methods and refined by least-squares procedures, respectively. Crystal structure diagrams were drawn with ORTEP-3 [41].

### 3.2. Photocatalyst Synthesis 

#### Copper (II) Complex

A modified method from the literature was followed [42] by reacting a solution of two equivalents of 1,10-phenanthroline in ethanol–H_2_O (90:10) solution with one equivalent of CuCl_2_.H_2_O under reflux for 3 h. The solid precipitate formed upon completion was filtered off, and the filtrate was kept at room temperature and allowed to evaporate slowly, yielding high-quality greenish-purple crystals suitable for X-ray analysis. [Cu(phen)_2_Cl]: colour, purple; FT-IR (cm^−1^): 3355 v(OH), 3051 v(CH), 1628 v(C=N), 1584 v(C=N), and 485 v(Cu–N).

### 3.3. Schiff Bases

#### 3.3.1. Synthesis of (*E*)-1-(4-((4-methylbenzylidene)amino)phenyl)ethanone (**1**)

An experimental method from the literature [43] was adopted for the synthesis of the title compound using 4-methylbenzaldehyde (2 g, 16.64 mmol), 4′-aminoacetophenone (2.25 g, 16.64 mmol), and EtOH (30 mL). The resulting mixture was heated under reflux overnight and cooled; then, the light-yellow solid was separated, filtered off, and recrystallized from acetone. Yield, 2 g (50.6%). IR (KBr): ṽ = 2980 *v*(CH), 2896 *v*(CH) 1664 *v*(C=O), and 1582 *v*(C=N). ^1^H NMR (400 MHz, CDCl_3_) δ 8.40 (s, 1H), 8.00 (s, 2H), 7.81 (s, 2H), 7.26 (d, *J* = 29.9 Hz, 4H), 2.61 (s, 3H), 2.44 (s, 3H). ^13^C NMR (101 MHz, CDCl_3_) δ 197.09, 162.00, 157.02, 142.95, 134.95, 133.25, 129.64, 129.33, 120.89, 26.31, and 21.28. Anal. Calc. for C_16_H_15_NO: C, 80.98; H, 6.37; and N, 5.90; Found: C, 80.27; H, 6.50; and N, 5.77.

#### 3.3.2. Synthesis of (*E*)-3-((4-(dimethylamino)benzylidene)amino)phenol (**2**)

The experimental procedure employed for the synthesis of **1** was followed using 4-(dimethylamino)benzaldehyde (4.66 g, 31.23 mmol) and 3-aminophenol (3.41 g, 31.23 mmol) in EtOH (50 mL). The crude product obtained after filtration was recrystallized using acetone to produce a yellow solid. Yield, 4.96 g (66.21%). IR (KBr): ṽ = 3644 *v*(OH), 2986 *v*(CH), 2903 *v*(CH), 2827 *v*(CH), 1680 *v*(C=C), and 1582 *v*(C=N). ^1^H NMR (400 MHz, DMSO) δ 9.52 (s, 1H), 8.36 (s, 1H), 7.74 (d, *J* = 8.7 Hz, 2H), 7.18 (t, *J* = 8.1 Hz, 1H), 6.74 (d, *J* = 8.7 Hz, 2H), 6.66 (s, 3H), and 2.96 (s, 6H). ^13^C NMR (101 MHz, DMSO) δ 160.24, 158.56, 154.22, 152.77, 130.73, 130.24, 124.28, 112.60, 112.07, 111.89, 108.35, and 40.11. Anal. Calc. for C_15_H_16_N_2_O: C, 74.97; H, 6.71; N, 11.66; and O, 6.66. Found: C, 72.58; H, 7.12; and N, 11.29.

#### 3.3.3. Synthesis of (*E*)-4-((4-hydroxybenzylidene)amino)-1,5-dimethyl-2-phenyl-1H-pyrazol-3(2H)-one (**3**)

Briefly, 4-hydroxybenzaldehyde (3 g, 24.56 mmol) and 4-aminoantipyrine (4.99 g, 24.56 mmol) were reacted in EtOH (50 mL). On completion, a light-yellow solid was produced. Yield, 5.75 g (76.2%). IR (KBr): ṽ = 3581 *v*(OH), 3024 *v*(CH), and 1584 *v*(C=N). ^1^H NMR (400 MHz, DMSO) δ 9.98 (s, 1H), 9.56 (s, 1H), 7.71 (d, *J* = 7.2 Hz, 2H), 7.50 (d, *J* = 6.3 Hz, 2H), 7.45–7.28 (m, 3H), 6.91 (d, *J* = 7.2 Hz, 2H), 3.08 (s, 3H), and 2.46 (s, 3H). ^13^C NMR (101 MHz, DMSO) δ 160.44, 156.01, 152.51, 134.71, 129.99, 127.06, 124.57, 117.90, 116.34, 35.97, and 10.28. Anal. Calc. for C_18_H_17_N_3_O_2_: C, 70.34; H, 5.58; N, 13.67; and O, 10.41. Found: C, 63.64; H, 6.24; and N, 12.48.

#### 3.3.4. Synthesis of (*E*)-1,5-dimethyl-4-((4-methylbenzylidene)amino)-2-phenyl-1H-pyrazol-3(2H)-one (**4**)

The experimental procedure employed for the synthesis of (*E*)-4-((5-bromo-2-hydroxybenzylidene)amino)-1,5-dimethyl-2-phenyl-1*H*-pyrazol-3(2H)-one was used in the synthesis of the title compound using 4-methylbenzaldehyde (2 g, 16.64 mmol) and aminoantipyrine (3.38 g, 16.64 mmol) in EtOH (30 mL). Upon completion, a yellow solid was recovered through filtration. Yield, 4.04 g (79.6%). IR (KBr): ṽ = 2993 *v*(CH), 2923 *v*(CH), 1659 *v*(C=O), and 1577 *v*(C=N). ^1^H NMR (400 MHz, CDCl_3_) δ 9.76 (s, 1H), 7.78 (d, *J* = 7.4 Hz, 2H), 7.49 (t, *J* = 7.2 Hz, 2H), 7.42 (d, *J* = 7.5 Hz, 2H), 7.32 (t, *J* = 7.1 Hz, 1H), 7.24 (d, *J* = 7.5 Hz, 2H), 3.12 (s, 3H), and 2.44 (d, *J* = 31.3 Hz, 6H). ^13^C NMR (101 MHz, CDCl_3_) δ 161.09, 157.59, 152.24, 141.06, 135.38, 134.71, 129.62, 127.77, 124.57, 118.55, 36.03, 21.36, and 10.29. Anal. Calc. for C_19_H_19_N_3_O: C, 74.73; H, 6.27; N, 13.76; and O, 5.24. Found: C, 73.77; H, 7.02; and N, 13.72.

### 3.4. Hydrocarboxylation Reaction

The Schiff bases (0.86 mmol), catalyst, (100.57 mg, 0.17 mmol), BIH (76.26 mg, 0.34 mmol), excess CyNMe_2_ (2mL, 13.35mmol), MeCN (10 mL), and a magnetic stirring bar were added to an oven-dried 50 mL round-bottomed flask. The mixture was purged with N_2_ for 2 min to evacuate the dissolved gases, and then purged with CO_2_ for 5 min to saturate the solution with CO_2_. The mixture was placed under a 30 W blue LED light source with constant stirring at room temperature for 24 h. The method for the synthesis of 1,3-dimethyl-2-phenyl-2,3-dihydro-1H-benzo[*d*]imidazole (BIH) is provided in the Supplementary Data section.

### 3.5. Theoretical Studies

#### 3.5.1. DFT Calculations

Molecular orbital calculations were conducted with full-geometry optimization of the catalyst and synthesized compounds (Schiff bases and unnatural α-amino acids) using the semi-empirical MO-G PM6 with the aid of the SCIGRESS package from Fijitsu (SCIGRESS version FQ 3.5.0). Three-dimensional molecular electrostatic potential (3D MESP) maps were also obtained from the optimized structures [44,45]. The highest occupied molecular orbital (HOMO) and lowest unoccupied molecular orbital (LUMO) energies, which determine chemical reactivity [9,10,11,46,47,48,49], are presented in the Supplementary Data section. Discussions of the global reactivity descriptors, such as the ionization potential (I), electron affinity (A), chemical potential (μ), electronegativity (χ), global hardness (η), global softness (S), and global electrophilicity (ω) values [12,13,14,16], are also presented in the Supplementary Data section. The molecular electrostatic potential (MEP) [17], which displays the electron-rich and -deficient regions of a molecule, is also presented in the Supplementary Data section.

#### 3.5.2. Docking Calculations

Three-dimensional crystal structures of E. faecalis DHFR (4M7U) co-crystallized with nicotinamide adenine dinucleotide phosphate (NADPH) [50] and B-DNA dodecamer (1BNA) [51] were obtained from the protein databank. The protein preparation wizard in Schrödinger suite 2022-1 was used to prepare 4M7U and 1BNA for docking calculations by refining the bond orders, adding hydrogens, deleting water molecules beyond 5 Å, filling missing loops using Prime, and generating states using Epik at pH 7.4. Protein minimization was performed using an OPLS4 force field with the RMSD of the crystallographic heavy atoms kept at 0.30 Å. In the case of 4M7U, the binding site was revealed by selecting the co-crystallized ligand (NADPH) and then creating a receptor grid with a docking length of 20 Å [46].

The possible binding sites of the B-DNA dodecamer (1BNA) were examined using Site Map, where 15 site points were analysed and then the site maps were cropped at 4 Å from the nearest site point. Amongst the various possible sites, the site with a higher score was used as the target site to generate the standard receptor grid for docking [47]. Docking and calculations were executed in the extra precision (XP) mode of Glide. The ligands with the highest negative Glide scores had more binding affinity toward 4M7U and 1BNA. To determine the free energy of binding for compounds **1**–**8** and standard drugs in the respective complexes, post-docking energy minimization studies were performed using Prime molecular mechanics-generalized Born surface area (MM-GB/SA) in Schrödinger 2022-1. The energy for the minimized XP docked pose of ligand–receptor complexes was calculated using the OPLS4 force field and generalized Born/surface area (GB/SA) continuum VSGB 2.0 solvent model [48].

#### 3.5.3. ADMET Determination

Synthesized compounds **1**–**8**, ciprofloxacin, and trimethoprim were prepared by assigning the bond length, bond angle, and possible ionization states at pH 7, and then optimized using the Ligprep module of the Schrödinger 2022–1 molecular modelling platform with an OPLS4 force field. Calculating the Qikprop scores, minimized ligands (Ligprep output) were given as the input in the Qikprop module for the determination of the ADMET (absorption, distribution, metabolism, excretion, and toxicity) properties [49].

## 4. Conclusions

We developed the catalytic hydrocarboxylation of Schiff bases using a copper(II) complex ([Cu(phen)_2_Cl], photocatalyst) with carbon dioxide (CO_2_) to generate substituted amino acids under mild reaction conditions. The Cu(II) ion of [Cu(phen)_2_Cl] is linked to four nitrogen (N) atoms of two 1,10-phenanthrolines and one chlorine atom in a slightly distorted square pyramidal geometry. The crystal packing revealed several intra- and inter-molecular hydrogen bonding interactions. The low energy gap (0.54, 0.80, and 1.99 eV) determined via DFT suggested a higher charge transfer when activated. The Schiff bases were successfully hydrcarboxylated with CO_2_ using the Cu(II) photocatalyst. The hydrocarboxylation reaction was successful in the presence of Cu(II), with the desired product yields in the range of 45 to 60%. The irradiation of Cu^2+^ confirmed its ability to absorb light and subsequent excitation of Cu^2+*^, which cause electron loss (e^−^) and result in the formation of the Cu(I), as confirmed by the disappearance of d–d transitions in the UV-Vis spectra. Both Schiff bases and α-substituted amino acids presented comparable binding free energies with E. faecalis (4M7U) similar to those of reference drugs (ciprofloxacin and trimethoprim). Compounds **1**–**8** presented groove binding with a DNA dodecamer (1BNA). The docking studies predicted the ligand–protein interaction through the complex formation and binding sites of the target proteins.

## Figures and Tables

**Table 1:** Selected crystal data and details for the determination of the photocatalyst.

Compound	[Cu(phen)_2_Cl]
Empirical formula	C_24_H_16_ClCuN_4_
Formula weight	459.40
Estimated formula	C_28_H_28_Cl_2_CuN_4_O_2_
Estimated moiety formula	C_24_H_16_ClCuN_4_, Cl, 2(C_2_H_6_O)
Estimated formula weight	587.01
Crystal colour	Green–purple
Crystal system	Monoclinic
Space group	C2/c (No.15)
Temperature (K)	296
a, b, c (Å)	23.2998(6) 30.2646(8) 7.4844(2)
α, β, γ (⁰)	90, 97.789(1), 90
V (Å^3^)	5229.0(2)
Z	8
F(000)	1872
*ρ*_calc_ (g/cm^3^)	1.167
Radiation (Å)	Moka 0.71073
Dataset	30:31; −40:40; −9:9
Theta Min–Max (De)	1.3, 28.3
Nref, Npar	6494, 271
Crystal Size (mm)	0.06 × 0.34 × 0.54
Min. and Max. Resd. Dens. (e/Ang^3)	−0.28, 0.32
R, wR2, S	0.0364, 0.1120, 1.01

**Table 2:** Selected bond lengths and bond angles of [Cu(phen)_2_Cl].

	Bond Length Experimental (Å)		Bond angle Experimental (°)
Cu_1_-N_11_	2.1073(19)	N_11_-Cu_1_-N_12_	81.21(7)
Cu_1-_N_12_	1.9872(17)	N_11_-Cu_1_-N_21_	123.14(6)
Cu_1_-N_21_	2.0997(16)	N_12_-Cu_1_-N_21_	95.15(7)
Cu_1_-N_22_	1.9769(17)	N_12_-Cu_1_-N_22_	175.97(7)
Cu_1_-Cl_1_	2.3368(7)	Cl_1_-Cu_1_-N_11_	115.28(5)
		Cl_1_-Cu_1_-N_12_	91.24(5)

**Table 3:** Selective hydrogen-bond, Y—X···π ring, and π···π stacking interactions for the crystal structure of [Cu(phen)_2_Cl].

Interactions	D—H (Å)	H···A (Å)	D···A (Å)	D—H···A (º)	Y―X…π (Å)	π···π (Å)
C_21_-H_21_…Cl_1_^i^	0.93	2.76	3.648(3)	161		
Cu_1_-Cl_1_…Cg1 ^ii^					3.5854(10)	
Cg2…Cg3 ^iii^	0.93	2.58	3.498(7)	171		3.7006(13)
Cg4…Cg1 ^i^						3.6196(12)

i: ½−x,1/2−y,1−z; ii: x,y, − 1 + x; iii: x,1−y, −1/2 + z; Cg1 is the centroid of C21, C22, C22, C221, C211, and C212; Cg2 is the centroid of N12 and C121 to C125; Cg3 is the centroid of C11, C12, C122, C121, C111, and C112; and Cg4 is the centroid of N21 and C211 to C215.

**Table 4:** Schiff base synthesis and desired hydrocarboxylation products.

Schiff Base Synthesis (Protocol 1)	Visible Light Hydrocarboxylation (Protocol 2)
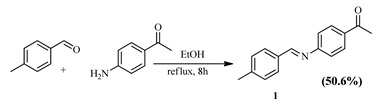	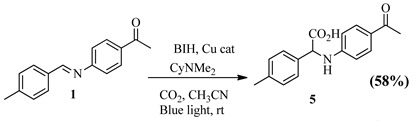
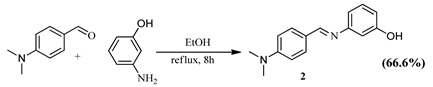	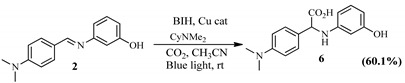
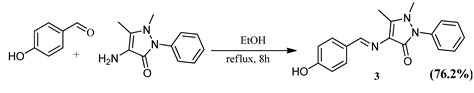	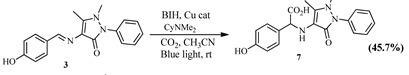
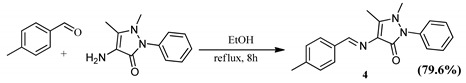	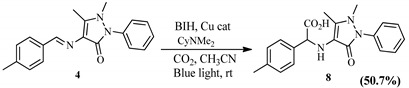

**Protocol 2**: Schiff Bases **1–4** (0.86 mmol), catalyst, (100.57 mg, 0.17 mmol), BIH (76.26 mg, 0.34 mmol), excess CyNMe_2_ (2 mL, 13.35 mmol), and MeCN (10 mL). Where BIH is 1,3-dimethyl-2-phenyl-2,3-dihydro-1H-benzo[d]imidazole, Cu cat is ([Cu(phen)_2_Cl, CO_2_ is Carbon dioxide, CyNMe_2_ is N,N-Dimethylcyclohexylamine, CH_3_CN is Acetonitrile EtOH is absolute ethanol and rt is room temperature.

**Table 5:** Frontier molecular orbital energies of the synthesized compounds.

Parameter	CAT	1	2	3	4	5	6	7	8
E_HOMO_ (eV)	−6.86	−9.04	−8.25	−8.55	−8.51	–9.29	–8.47	–9.02	–8.81
E_LUMO_ (eV)	−0.94	−1.12	–0.20	−0.42	−0.30	–0.77	–0.16	–0.82	−0.66
ΔE_gap_ (eV)	5.92	7.92	8.05	8.13	8.21	8.52	8.31	8.20	8.15
I (eV)	6.86	9.04	8.25	8.55	8.51	9.29	8.47	9.02	8.81
A (eV)	0.94	1.12	0.20	0.42	0.30	0.77	0.16	0.82	0.66
μ (eV)	−3.90	–5.08	–4.23	–4.49	–4.41	−5.03	−4.31	–4.92	−4.74
χ (eV)	3.90	5.08	4.23	4.49	4.41	5.03	4.31	4.92	4.74
ƞ (eV)	2.96	3.96	4.03	4.07	4.11	4.26	4.16	4.10	4.08
S (eV)	0.34	0.25	0.25	0.25	0.24	0.24	0.24	0.24	0.25
ω (eV)	22.51	3.26	2.22	2.48	2.37	2.97	2.23	2.95	2.75

CAT = ([Cu(phen)_2_Cl].

**Table 6:** Energies of Schiff bases (**1**–**4**) and α -unsaturated amino carboxylic acids (**5**–**8**) with enzyme E. faecalis DHFR (4M7U) and B-DNA dodecamer (1BNA).

	G−Score		E−Model		Ligand Efficiency	
Receptor/DNA	4M7U	1BNA	4M7U	1BNA	4M7U	1BNA
**1**	−4.759	−3.434	−41.752	−30.420	−0.264	−0.191
**2**	−4.557	−5.815	−39.811	−52.597	−0.252	−0.178
**3**	−4.891	−3.976	−47.146	−33.735	−0.213	−0.173
**4**	−3.255	−4.995	−29.960	−53.619	−0.140	−0.067
**5**	−5.902	−2.420	−59.663	−21.008	−0.281	−0.122
**6**	−5.931	−4.013	−58.006	−27.247	−0.280	−0.129
**7**	−5.656	−2.369	−62.981	−27.070	−0.217	−0.130
**8**	−5.626	−2.603	−63.009	−26.668	−0.216	−0.112
**Trimethoprim**	−5.837	−4.289	−45.603	−32.005	−0.248	−0.160
**Ciprofloxacin**	−4.228	−3.369	−45.719	−27.134	−0.132	−0.136

**Table 7:** Binding free energy components for 4M7U-ligand complexes calculated by MM-GBSA analysis.

Ligands	Enterococcus Faecalis 4M7U (kcal/mol)							
	ΔG_Bind_	ΔG_Coul_	ΔG_cov_	ΔG_Hbond_	ΔG_Pack_	ΔG_lipho_	ΔG_Solv_GB_	ΔG_VdW_
***Schiff Bases***								
**1**	−39.21	−15.43	1.73	−0.59	0.00	−15.39	22.13	−31.67
**2**	−29.23	−7.95	0.25	−0.53	0.00	−12.53	18.34	−26.80
**3**	−39.21	−22.95	6.04	−1.77	−0.08	−14.51	24.14	−30.08
**4**	−39.73	−18.96	2.22	−0.63	−0.05	−15.19	24.49	−31.61
**α-unsaturated aminocarboxylic acids**								
**5**	−37.87	−16.63	3.41	−3.06	−0.12	−11.16	22.98	−33.29
**6**	−37.64	−23.74	1.67	−2.04	−0.19	−12.64	31.33	−32.02
**7**	−41.45	−29.35	10.04	−2.27	0.00	−14.79	29.71	−34.79
**8**	−39.27	−23.82	10.13	−1.67	0.00	−15.51	29.57	−37.97
**Control**								
**Trimethoprim**	−36.94	−34.03	2.39	−3.18	−0.97	−8.45	30.82	−23.51
**Ciprofloxacin**	−31.72	−40.54	0.42	−2.22	−1.27	−9.99	50.27	−28.39

ΔG Bind: Binding free energy; ΔG Coul: Coulomb or electrostatic interaction energy; ΔG_cov:_ Covalent bonding correction, ΔG_Hbond:_ Hydrogen bonding correction_,_ ΔG_Pack_: π–π packing correction, ΔG_lipho_ Lipophilic interaction energy, ΔG Solv_GB: Generalized Born electrostatic solvation energy, ΔG vdW: Van der Waals interaction energy.

**Table 8:** Hydrogen bonding and mixed π-interactions of Schiff bases (**1**–**4**) and α-unsaturated amino carboxylic acids (**4**–**8**) with Enterococcus faecalis enzyme (4M7U) and B-DNA dodecamer (1BNA).

		Enterococcus Faecalis (PDB id: 4M7U) with Interacting Residues		B-DNA Dodecamer (PDB id: 1BNA) with Interacting Nucleotides	
Entry		Hydrogen Bond (Å)	π-Interactions (Å)	Hydrogen Bond (Å)	π-π Stacking (Å)
Schiff bases					
**1**		SER_100_ (1.86)	-	DA_6_ (2.27)	DA_5_ (5.23), DG_4_ (4.80)
**2**		GLY_18_ (1.74)	GLY18 (2.30) π-alkyl	DA_18_ (2.37)	DC_3_ (5.21)
**3**		ALA_45_ (2.69), SER_65_ (2.05), (1.80)	ARG44 (6.26) π-cation	DT_19_ (1.89), DC_3_ (1.97)	DG_4_ (5.00)
**4**		VAL_101_ (2.73), GLY_99_ (2.68)	-	DG_4_ (2.75)	DG_4_ (5.14), DC_3_ (5.24)
α-unsaturated aminocarboxylic acids					
**5**		VAL_102_ (2.04), ARG44 (2.60), SER_65_ (1.88), THR64 (2.07)	SER65 (2.38), (2.29) π-alkyl	DC_3_ (1.65)	**-**
**6**		GLU105 (1.75), SER65(2.63)	-	DC_3_ (2.07), DC_21_ (2.74), DT_19_ (1.78)	**-**
**7**		ALA45 (1.80), THR46 (2.60). GLY99 (2.60), THR126 (1.90)	ARG44 (6.52) π-cation	DC_3_(1.64), DC_21_ (2.74)	-
**8**		ALA45 (1.83), THR46 (2.60)		DC_3_ (1.69), DC_21_ (2.33)	-
Control					
**Trimethoprim**		SER_65_ (2.04), ARG_44_ (1.93)	ARG_44_ (5.78) π-cation	DC_3_ (1.91), DG_4_ (1.92)	-
**Ciprofloxacin**		ASP125 (1.80), VAL101 (2.11), THR46 (2.20)	-	DG_4_ (2.24), DA_18_ (2.55), DA_17_ (1.60)	DA_18_ (4.74)

**Table 9:** ADMET properties of the studied compounds.

Entry	1	2	3	4	5	6	7	8	Trimethoprim	Ciprofloxacin
Mw	237.30	240.31	307.35	305.38	283.33	286.33	353.38	351.41	290.32	331.34
#stars	0	0	0	0	0	0	0	0	0	1
WPSA	0	0	0	0	0	0	0	0	0	31.43
Volume (A^3^)	889.07	879.93	1005.64	1051.19	969.90	950.591	1151.07	1182.23	930.09	1013.05
QPpolrz (A^3^)	29.70	28.86	34.57	36.62	31.86	30.32	40.02	41.58	27.50	34.40
EA (eV)	0.94	0.46	0.54	0.49	0.39	−0.02	0.27	0.27	−0.092	0.76
QplogPoct	11.42	11.97	10.71	14.86	16.12	17.09	23.09	21.15	17.52	17.79
QplogPw	5.52	6.60	10.16	7.81	10.52	11.43	15.87	13.31	12.11	9.94
QplogPo/w	3.59	3.55	2.79	3.82	2.98	2.69	0.25	1.23	0.91	0.280
QplogS	−4.17	−4.21	−3.81	−4.44	−4.06	−3.32	−4.10	−4.85	−2.85	−3.79
QPPCaco (nm/s)	2670.97	2535.63	1248.80	4244.01	117.72	88.16	10.26	36.48	2396.80	12.98
#metab	1	2	2	2	4	6	4	4	5	0
%Human Oral Absor	100	100	100	100	81.46	77.53	46.52	62.09	78.08	48.51
PSA	39.24	35.097	62.99	40.68	85.21	87.30	112.60	90.10	98.46	98.88
Rule of 3	0	0	0	0	0	0	1	0	0	1
Rule of 5	0	0	0	0	0	0	0	0	0	0

## Data Availability

Data is contained within the article and  Supplementary Material.

## Supplementary Materials

The following supporting information can be downloaded at: https://www.mdpi.com/article/10.3390/ph15101240/s1. CCDC 2204542 contains the crystallographic data of [Cu(phen)_2_Cl]. These data can be obtained freely from the Cambridge Crystallographic Data Centre via www.ccdc.cam.ac.uk/structures (accessed on 6 September 2022). Reference [52] is cited in the Supplementary Materials.
