# Supplementary material for: Cu(II)-Catalysed Hydrocarboxylation of Imines Utilizing CO2 to Synthesize α-Unsaturated Aminocarboxylic Acids
Source: Pharmaceuticals (Basel). 2022 Oct 9;15(10):1240. doi: 10.3390/ph15101240 (PMC9610938; doi:10.3390/ph15101240)
Supplement: Supplementary file 1 [file pharmaceuticals-15-01240-s001.zip › pharmaceuticals-1921812-supplementary.pdf]

Cu(II) catalysed hydrocarboxylation of imines utilizing CO_2 to synthesize α-unsaturated aminocarboxylic acids

Allen T. Gordon[a*], Eric C. Hosten[a], Adeniyi S. Ogunlaja[a*]

Department of Chemistry, Nelson Mandela University, Gqeberha (Port Elizabeth), South Africa

Supplementary data section

Synthesis of 1,3-dimethyl-2-phenyl-2,3-dihydro-1H-benzo[d]imidazole (BIH)

BIH was synthesized from a modified method based on the literature [1] in a twostep pathway. Firstly, 2-phenylbenzimidazole (3 g, 15.45 mmol) in a round bottomed flask was treated with methyl iodide (8 g, 56.36 mmol) in MeOH (20 mL) and NaOH (0.64 g, 16 mmol). The reflux apparatus was connected then the mixture heated at 110 °C for 24 h to give a faint yellow precipitate, which was filtered off, washed with excess water, and dried to give $BIH^{+}I^{-}$, Yield (3.2 g, 58.9%). The obtained product was used without further purification in the second step. Secondly, $BIH^{+}I^{-}$ (1.5 g, 4.28 mmol) was suspended in MeOH (57 mL) and the mixture was degassed using N_2 and cooled to 0°C in an ice-water bath. Then $NaBH_4$ (405 mg, 10.7 mmol) in small portions was added. After completion of borohydride addition, the reaction mixture was left to warm to RT and stirred for 3 h under inert atmosphere. The reaction mixture was concentrated in vacuo, then 50 mL of water added and extracted by ether (3 x 25 mL). The organic extract was washed with water (10 mL), brine (20 mL), dried over Na_2SO_4, filtered, and evaporated in vacuo to dryness. The residue was dissolved in 2:1 EtOH-H_2O (40 mL) at 75°C, filtered through cotton wool while hot and then slowly cooled to room temperature. The crystals were filtered and dried in high vacuum.

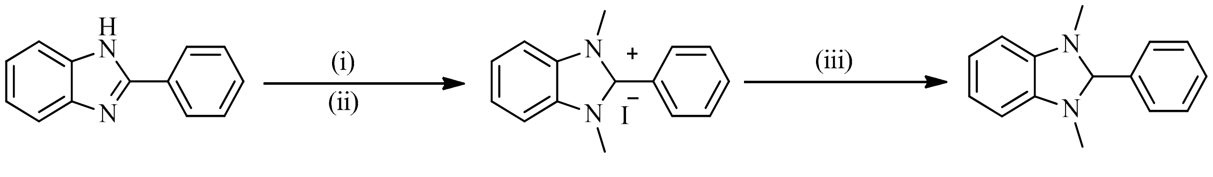

Reagents and conditions: (i) MeI, NaOH (ii) MeOH, Δ, H_2O (iii) $NaBH_4$, N_2, EtOH

Scheme S1: Synthesis of 1,3-dimethyl-2-phenyl-2,3-dihydro-1H-benzo[d]imidazole (BIH)

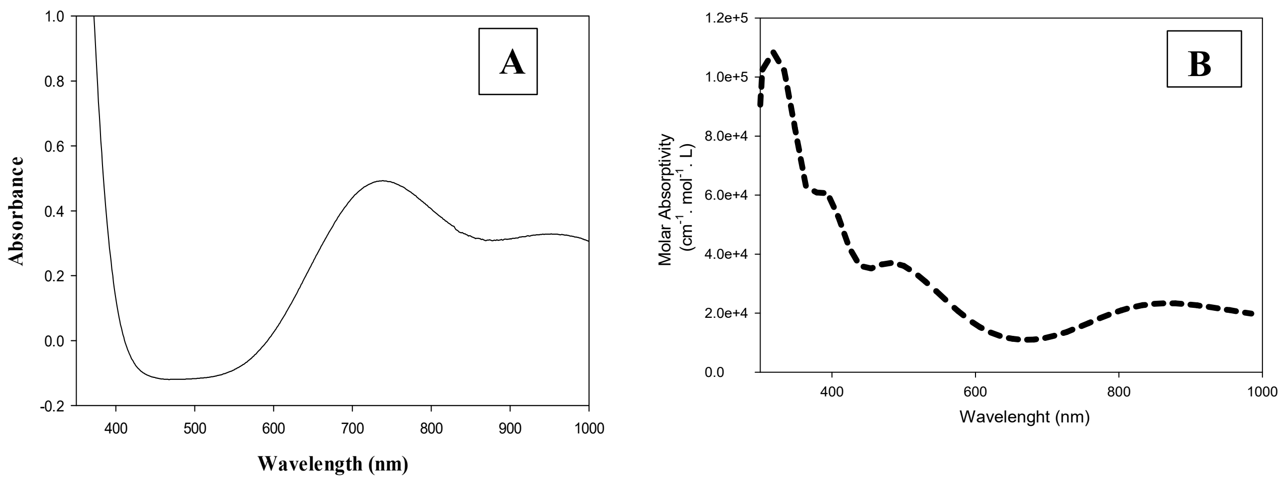

Figure S1: UV-Vis spectrum of **CAT**, (**A**) Experimental and (**B**) Theoretical (at B3LYP/LanL2DZ)

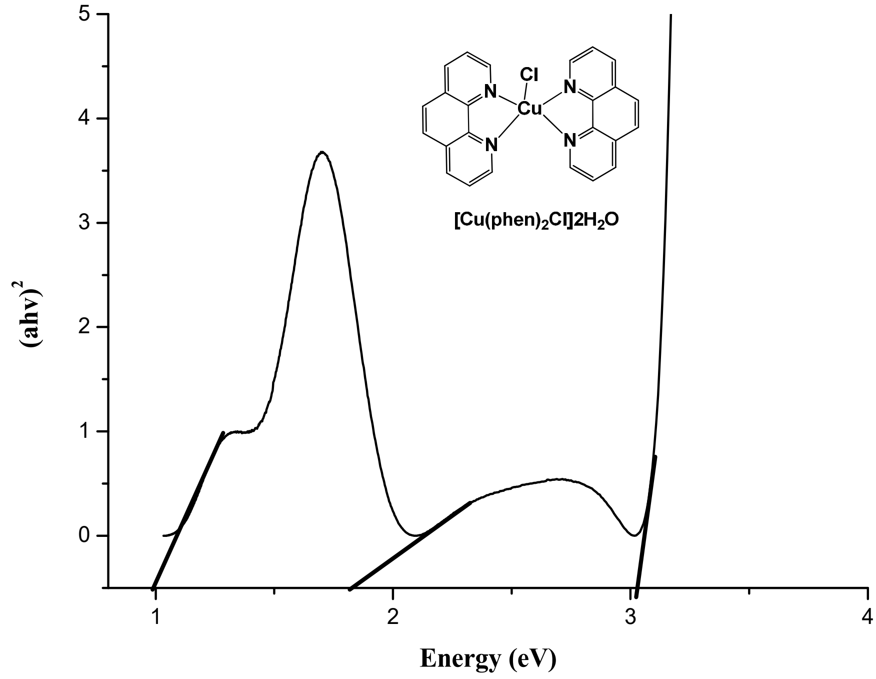

Figure S2: E_g value for **CAT** obtained from a UV-Vis spectrum

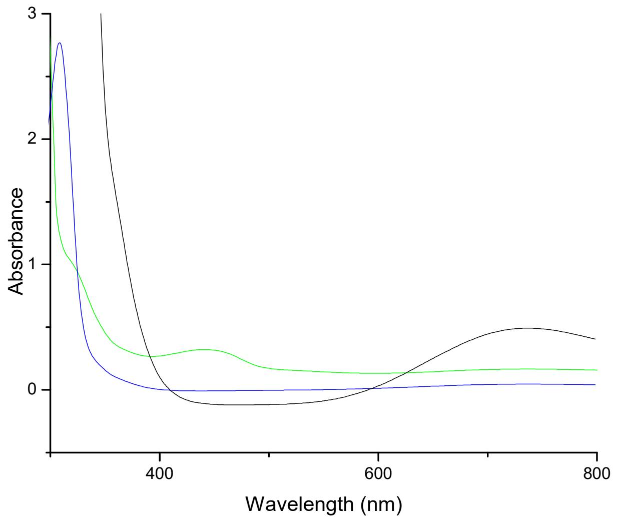

Re: (Black cat before irradiation), blue (cat +BIH) and yellow (Cat+CyNMe2)

Figure S3: UV-Vis spectrum of photocatalyst and additives (BIH and CyNMe2)

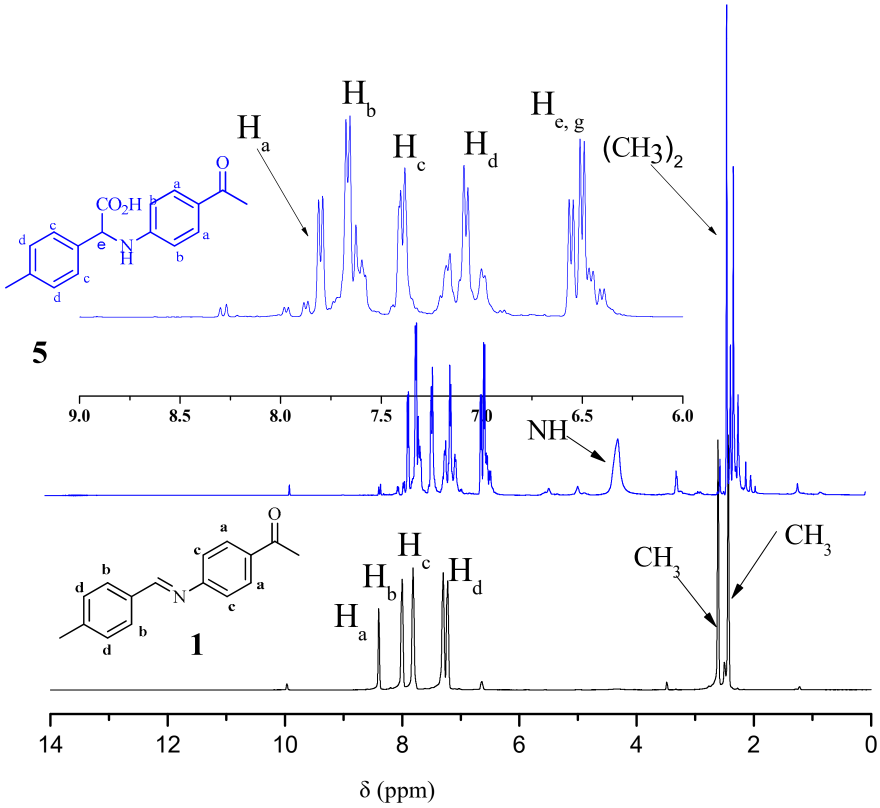

Figure S4:^{1}H NMR spectrum of **1** and product **HYD1** obtained after irradiation with CO_2

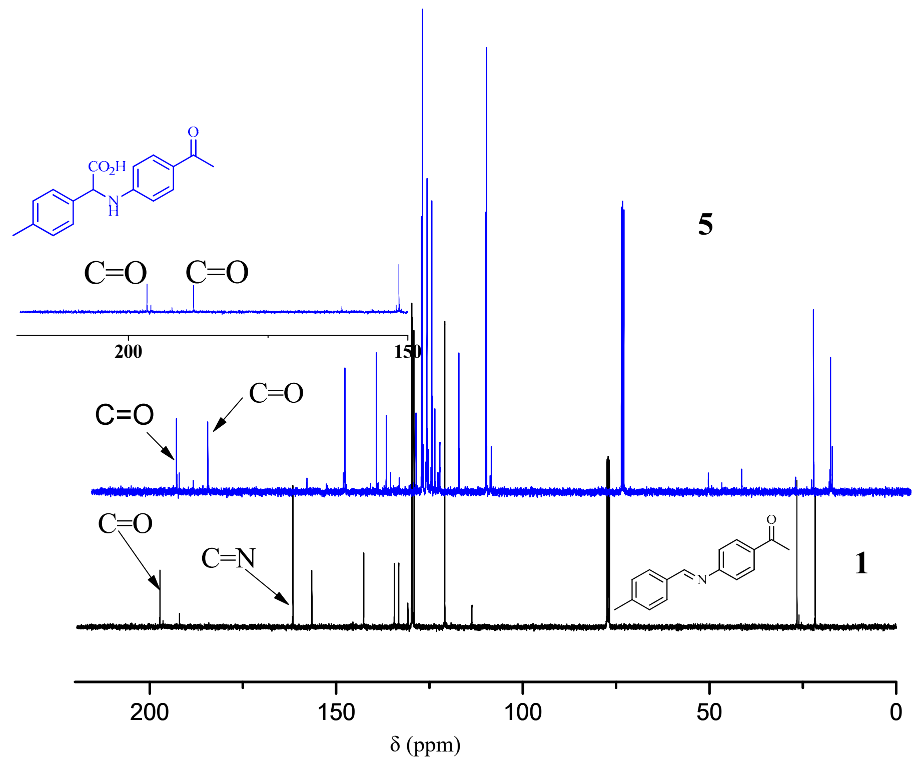

Figure S5: ^{13}C NMR spectrum of **1** and product **HYD1** obtained after irradiation with CO_2

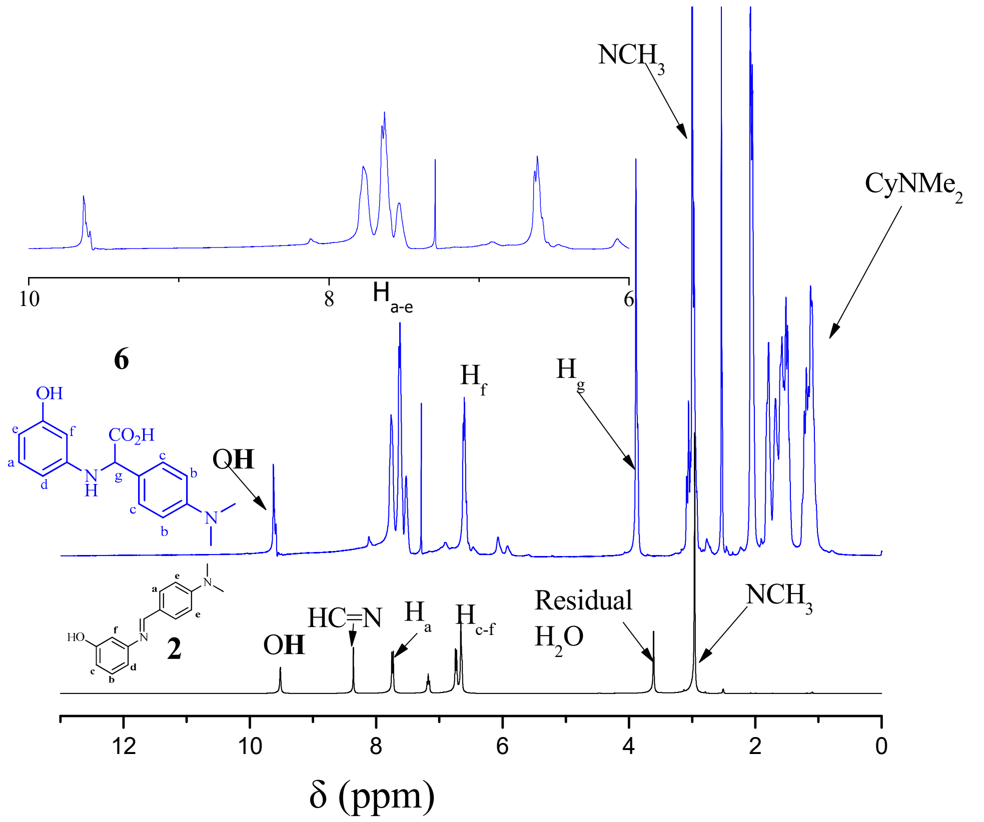

Figure S6: ^{1}H NMR spectrum of **2** and product **6** obtained after irradiation with CO_2

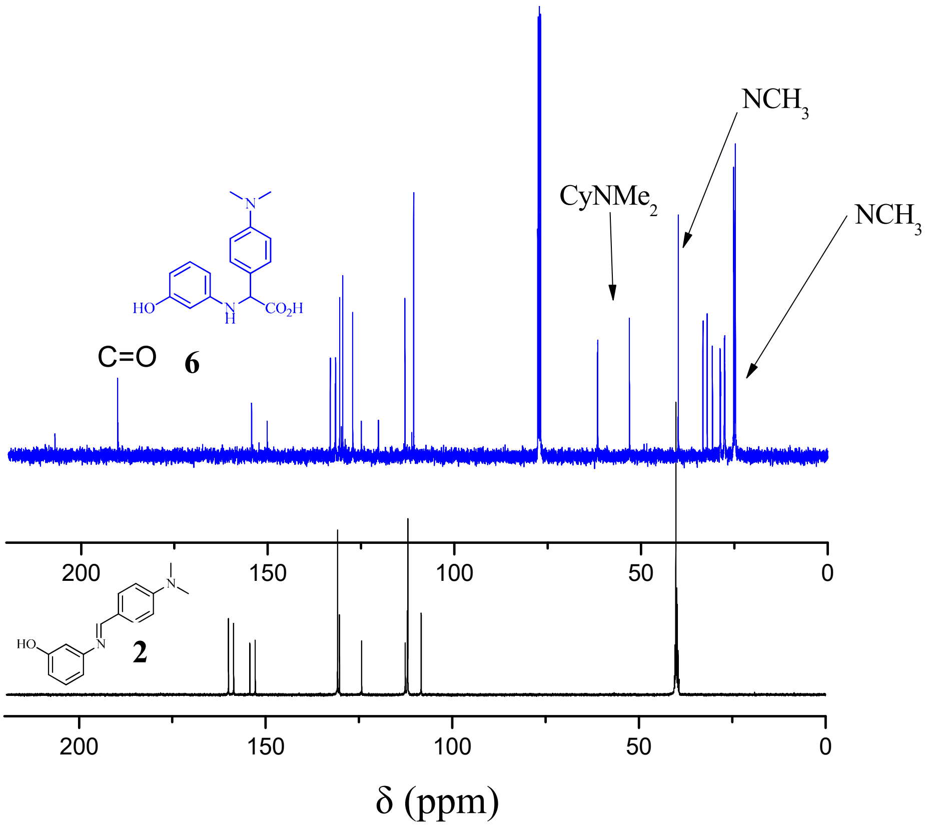

Figure S7: ^{13}C NMR spectrum of **2** and product **6** obtained after irradiation with CO_2

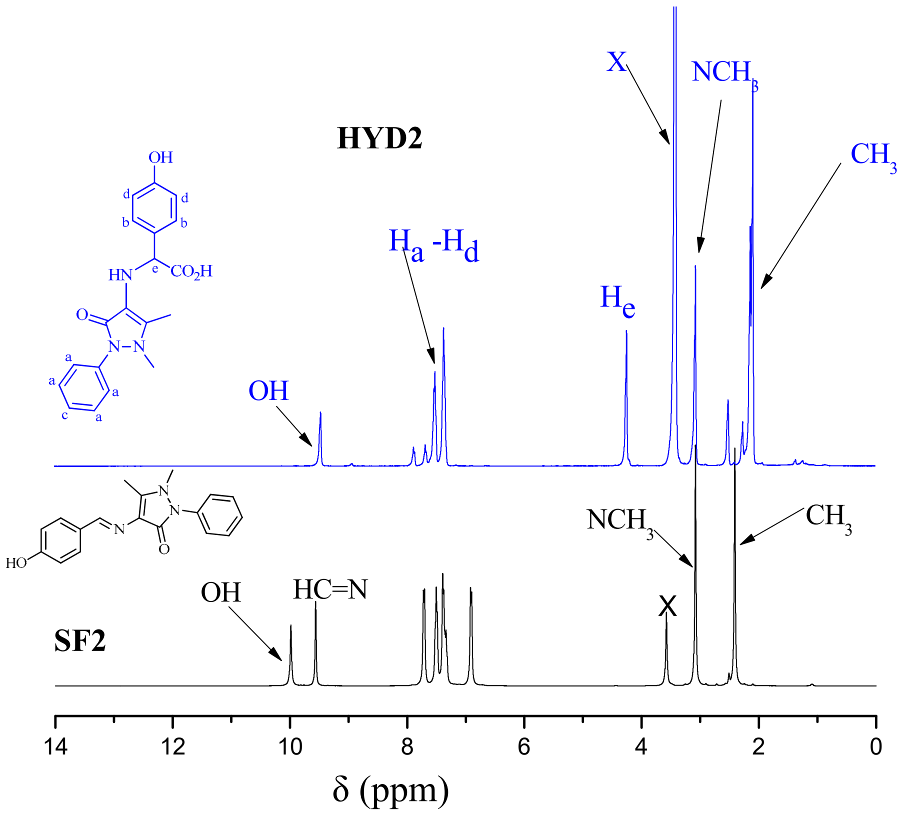

Figure S8: ^{1}H NMR spectrum of **3** and product **7** obtained after irradiation with CO_2

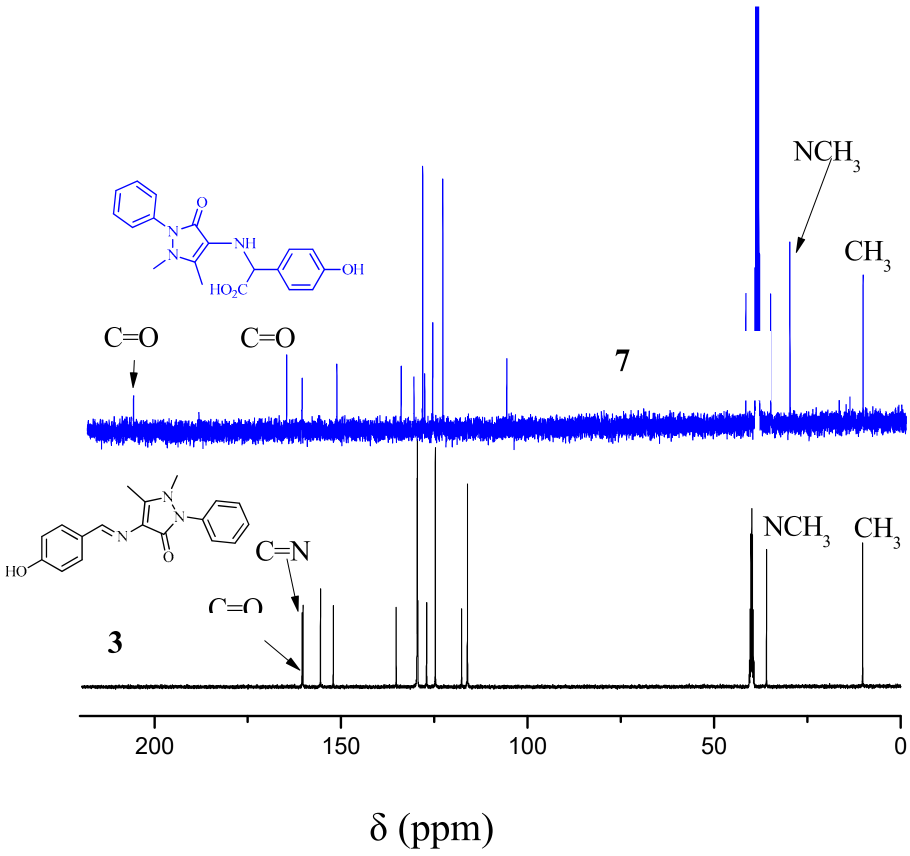

Figure S9: ^{13}C NMR spectrum of **3** and product (**7**) obtained after irradiation with CO_2

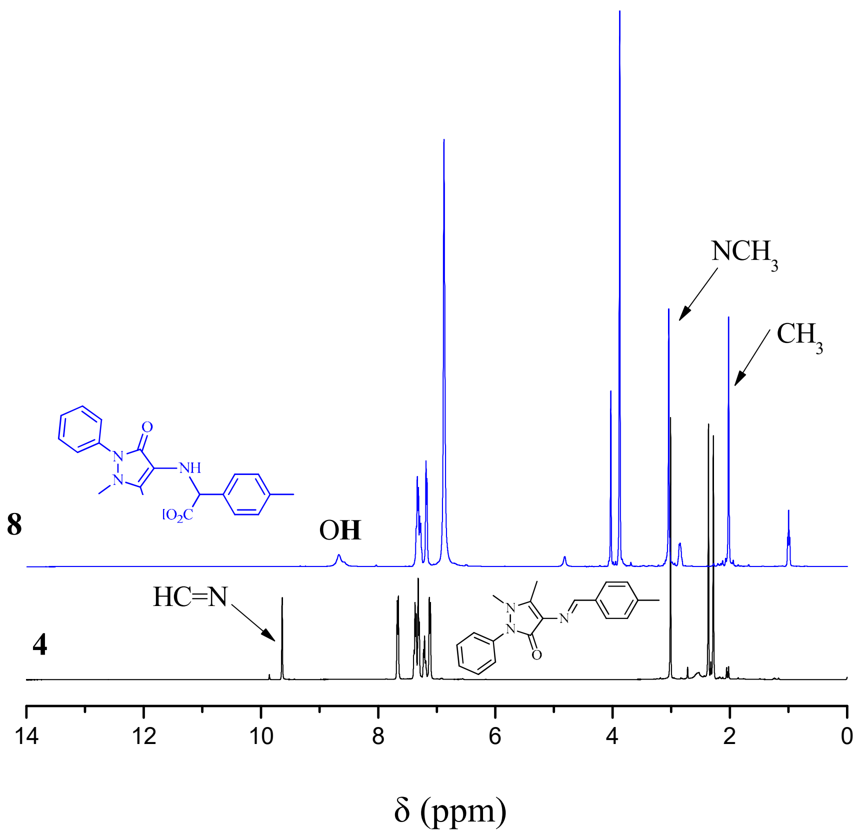

Figure S10: ^{1}H NMR spectrum of **4** and product **8** obtained after irradiation with CO_2

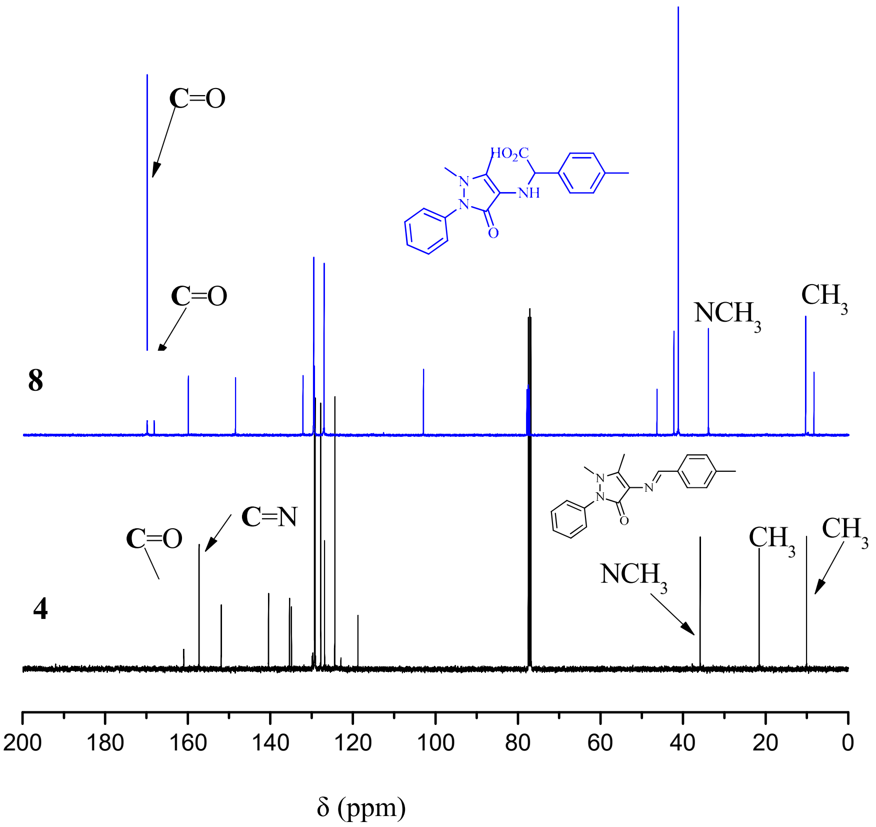

Figure S11: ^{13}C NMR spectrum of **4** and product **8** obtained after irradiation with CO_2

Molecular Docking

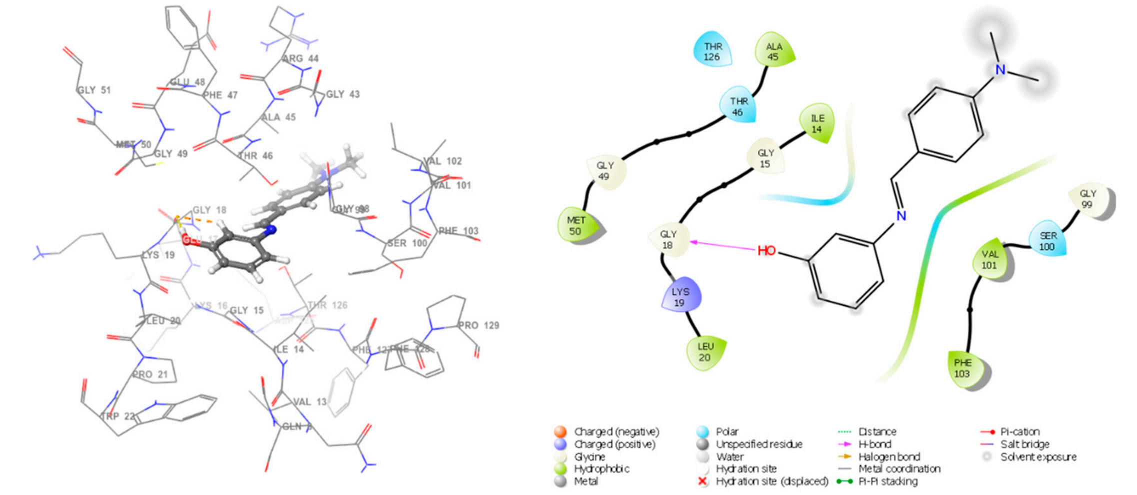

Figure S12: The 3D binding model of **2** with 4M7U (**A**), 2D diagram of **2** and interacting residues (**B**)

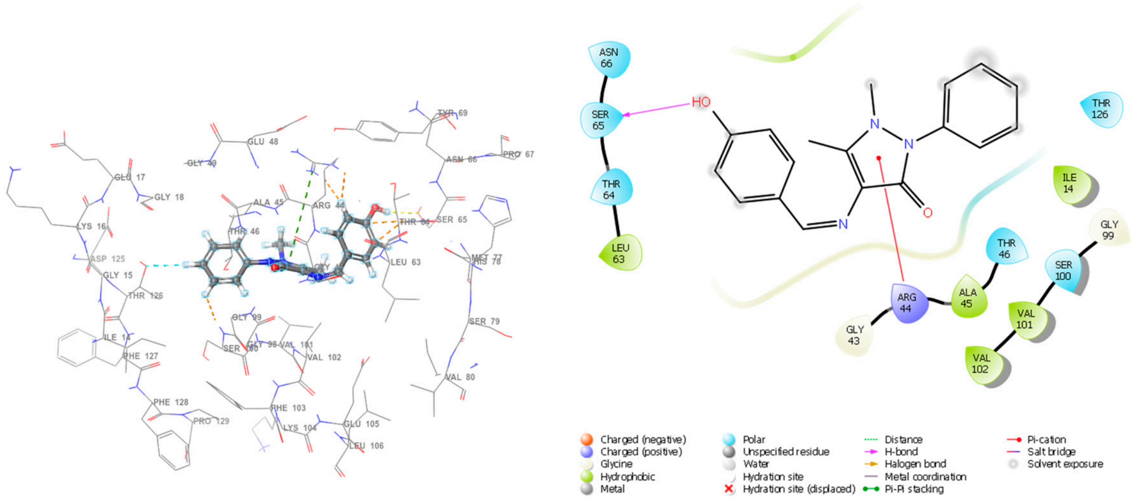

Figure S13: The 3D binding model of **3** with 4M7U (**A**), 2D diagram of **3** and interacting residues (**B**)

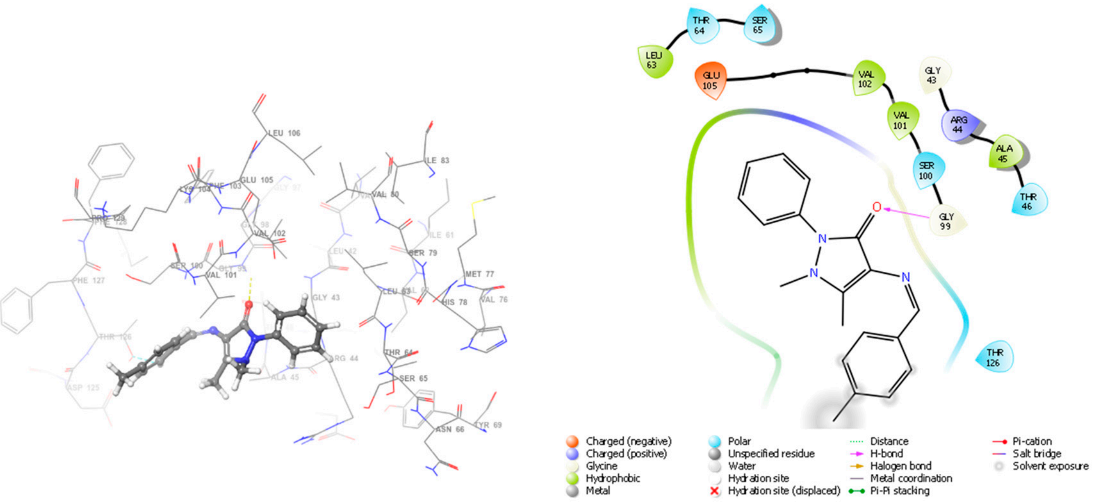

Figure S14: The 3D binding model of **4** with 4M7U (**A**), 2D diagram of **4** and interacting residues (**B**)

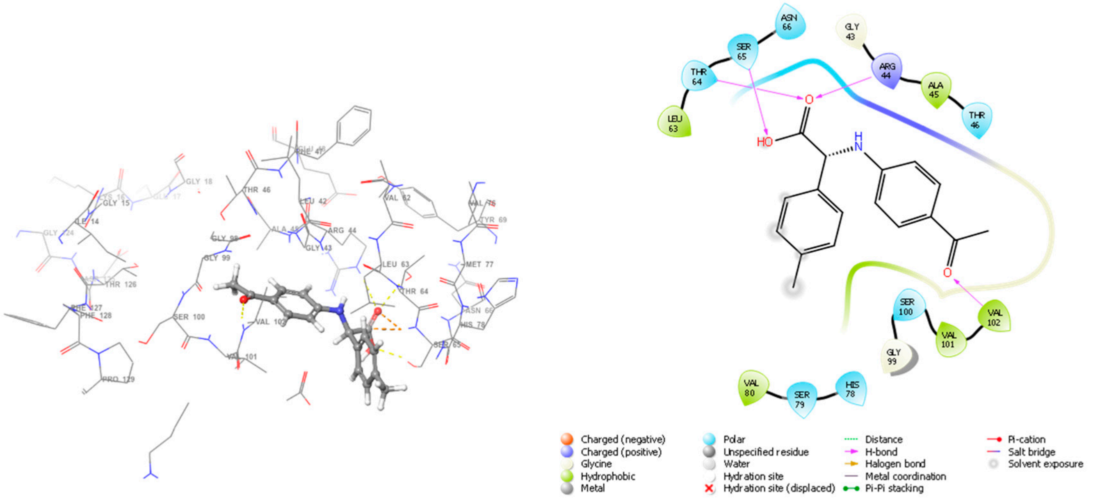

Figure S15: The 3D binding model of **5** with 4M7U (**A**), 2D diagram of **5** and interacting residues (**B**)

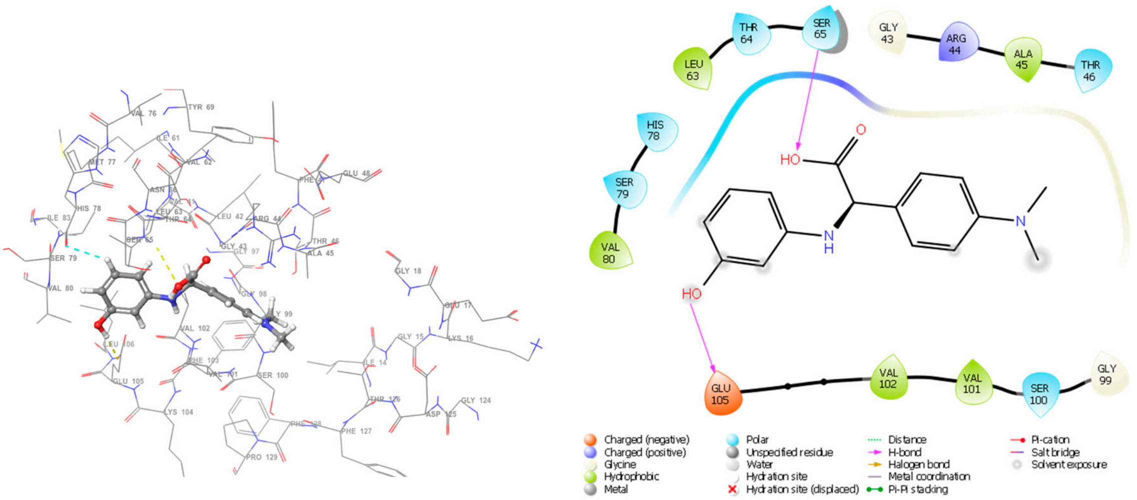

Figure S16: The 3D binding model of **6** with 4M7U (**A**), 2D diagram of **6** and interacting residues (**B**)

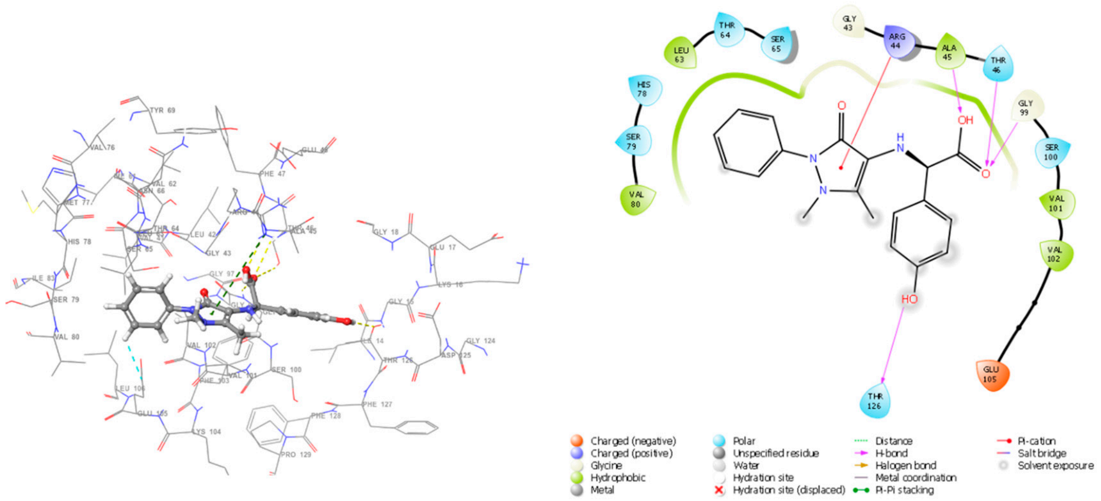

Figure S17: The 3D binding model of **7** with 4M7U (**A**), 2D diagram of **7** and interacting residues (**B**)

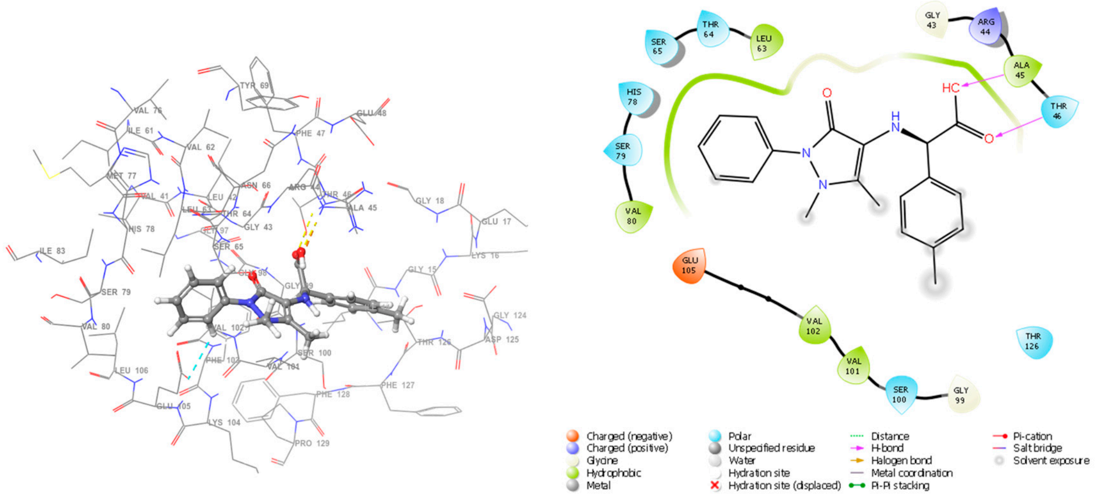

Figure S18: The 3D binding model of **8** with 4M7U (**A**), 2D diagram of **8** and interacting residues (**B**)

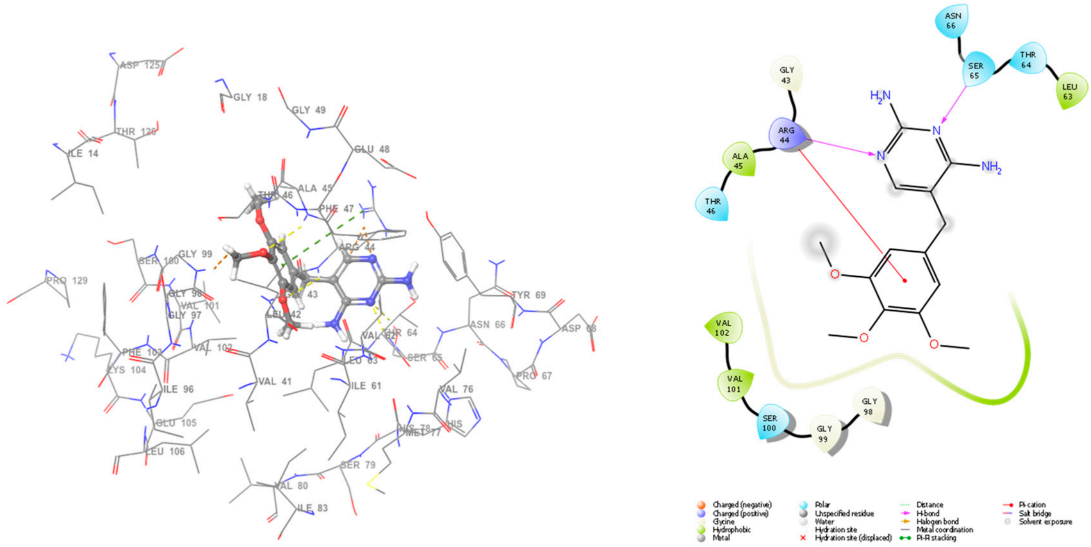

Figure S19: The 3D binding model of **Trimethoprim** with 4M7U (**A**), 2D diagram of **Trimethoprim** and interacting residues (**B**)

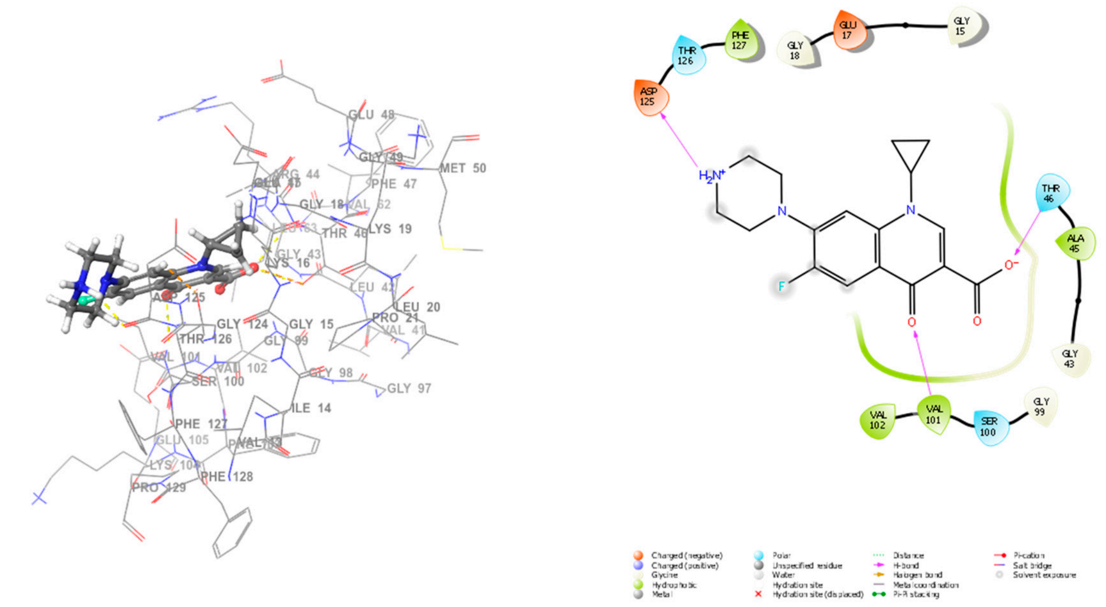

Figure S20: The 3D binding model of **Ciprofloxacin** with 4M7U (**A**), 2D diagram of **Ciprofloxacin** and interacting residues (**B**)

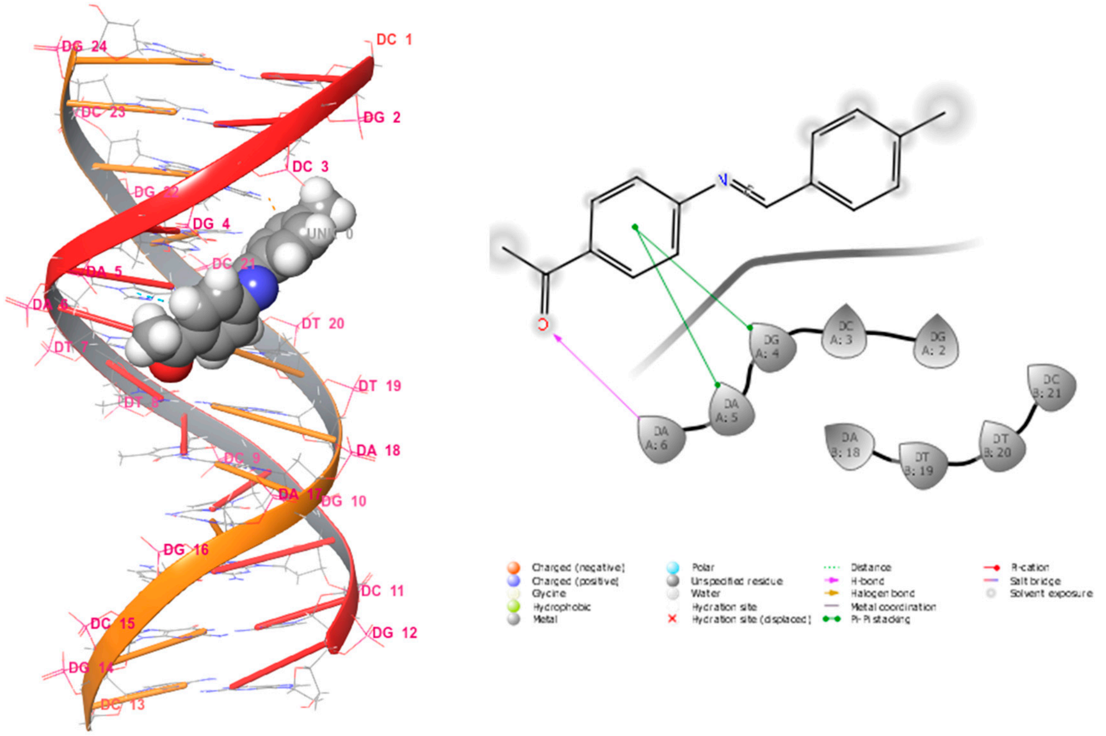

Figure S21: The 3D binding model of **1** with B-DNA dodecamer (1BNA) (**A**), 2D diagram of **1** and interacting residues (**B**)

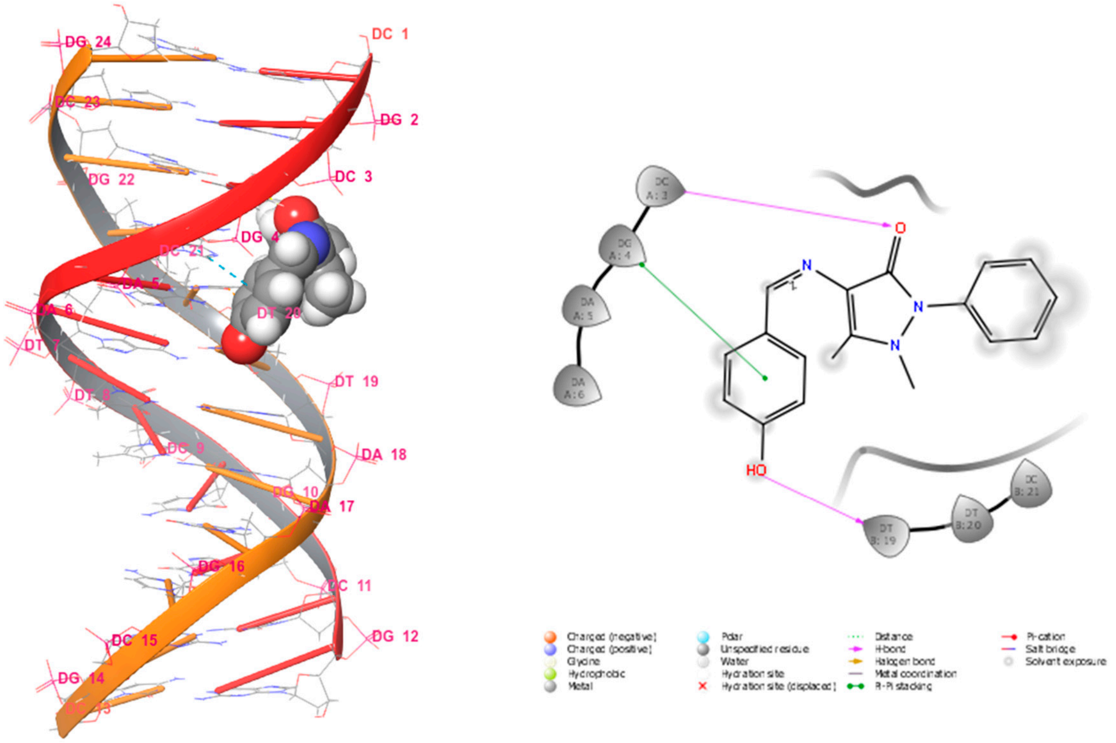

Figure S22: The 3D binding model of **3** with B-DNA dodecamer (1BNA) (**A**), 2D diagram of **3** and interacting residues (**B**)

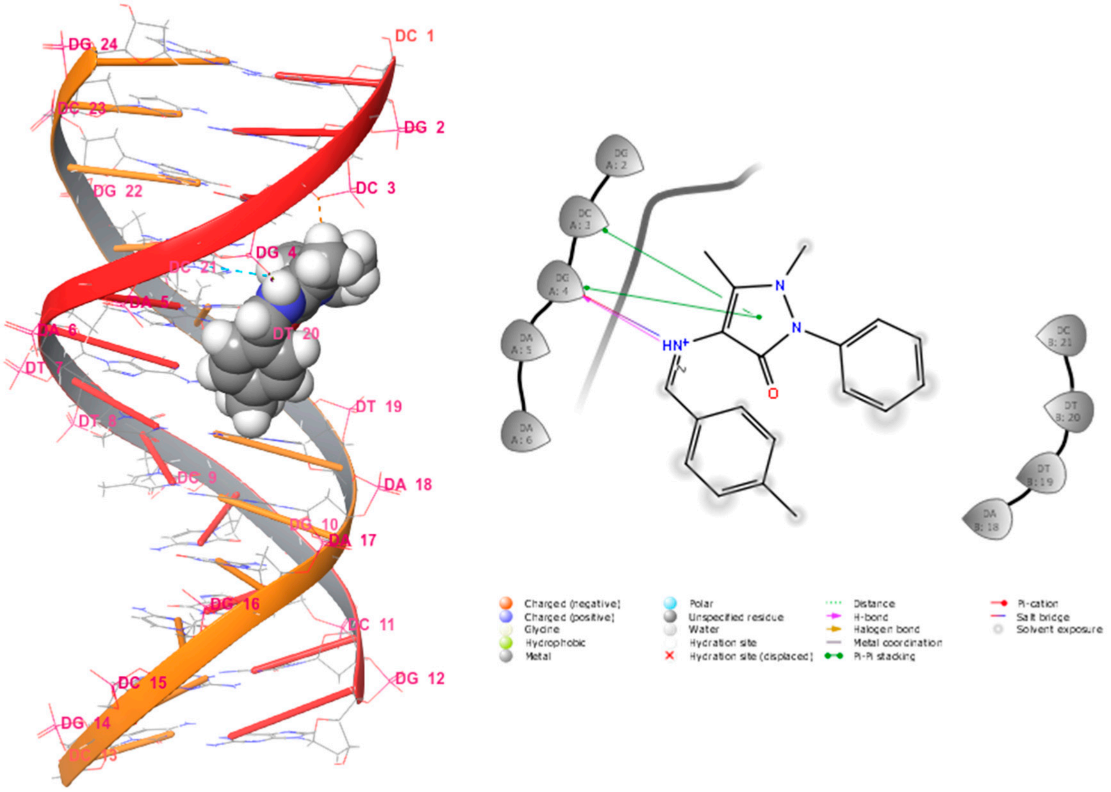

Figure S23: The 3D binding model of **4** with B-DNA dodecamer (1BNA) (**A**), 2D diagram of **4** and interacting residues (**B)**

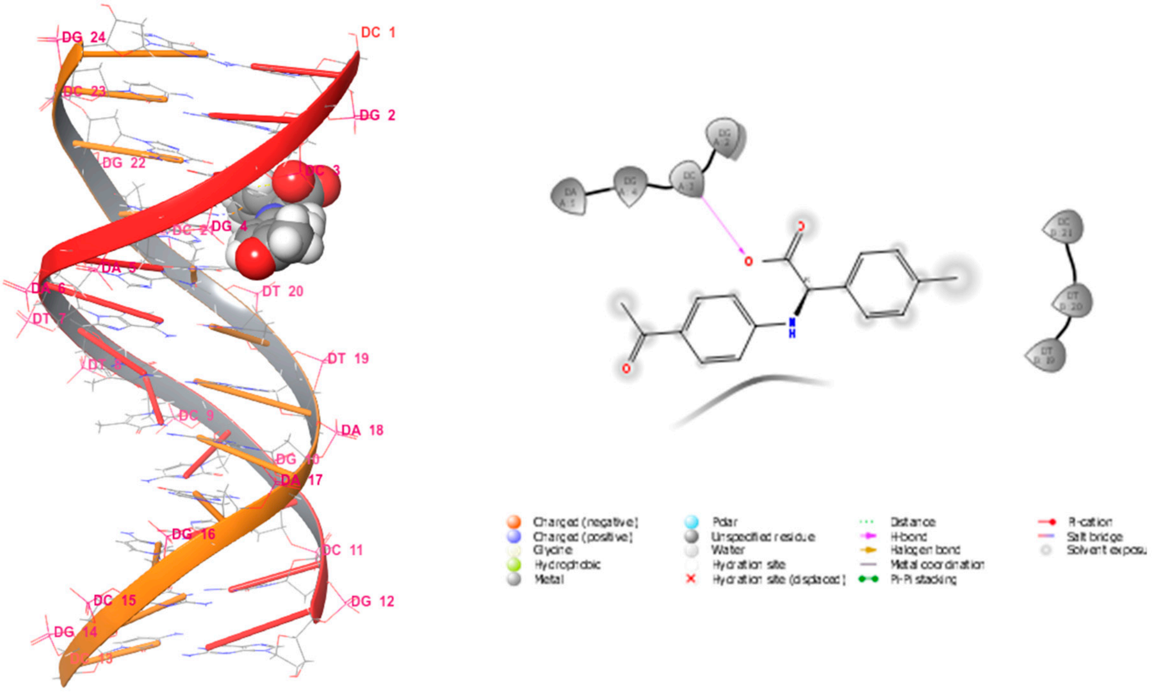

Figure S24: The 3D binding model of **5**with B-DNA dodecamer (1BNA) (**A**), 2D diagram of **1** and interacting residues (**B)**

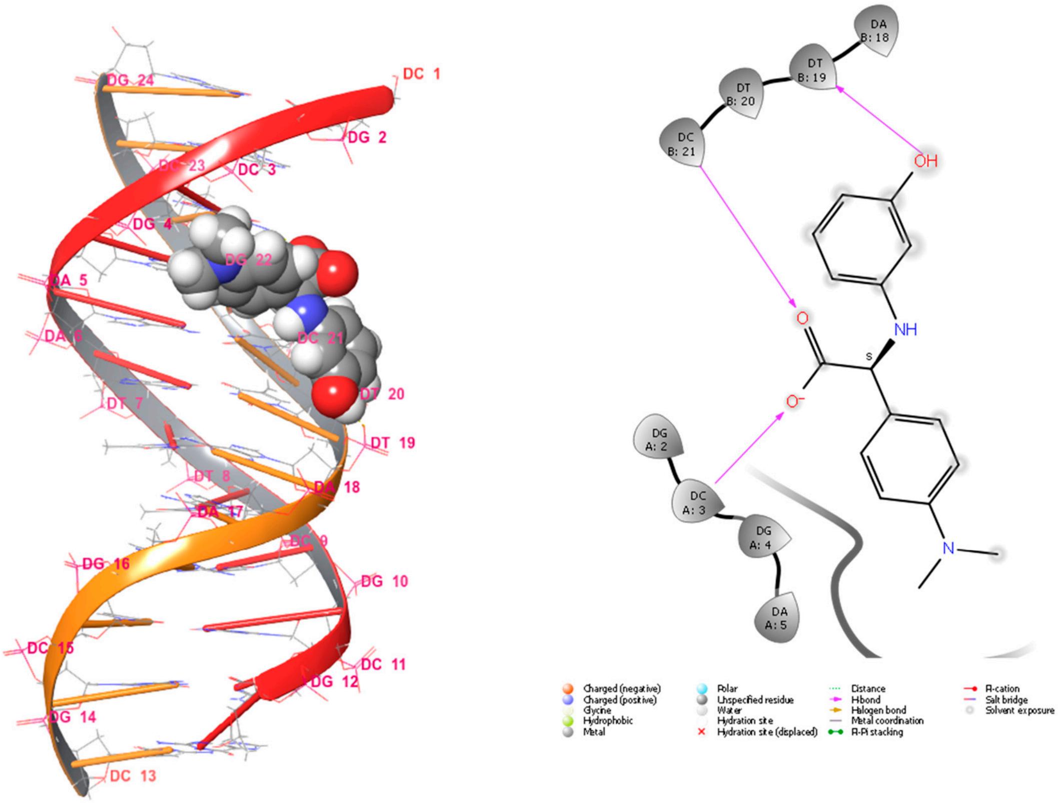

Figure S25: The 3D binding model of **6** with B-DNA dodecamer (1BNA) (**A**), 2D diagram of **6** and interacting residues (**B**)

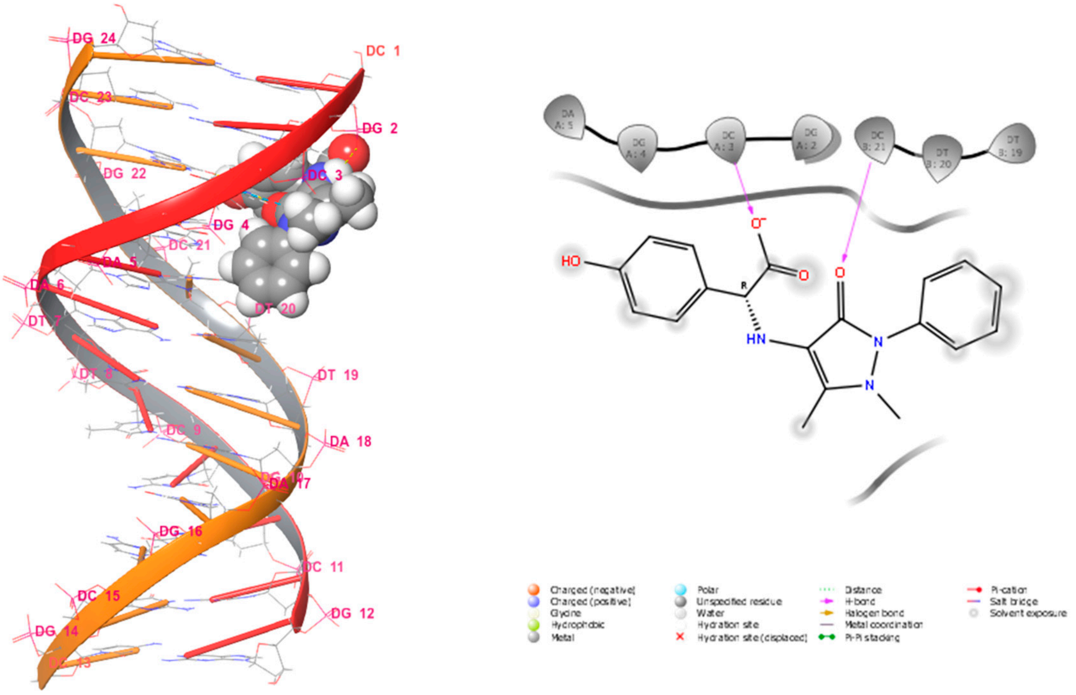

Figure S26: The 3D binding model of **7** with B-DNA dodecamer (1BNA) (**A**), 2D diagram of **7** and interacting residues (**B**)

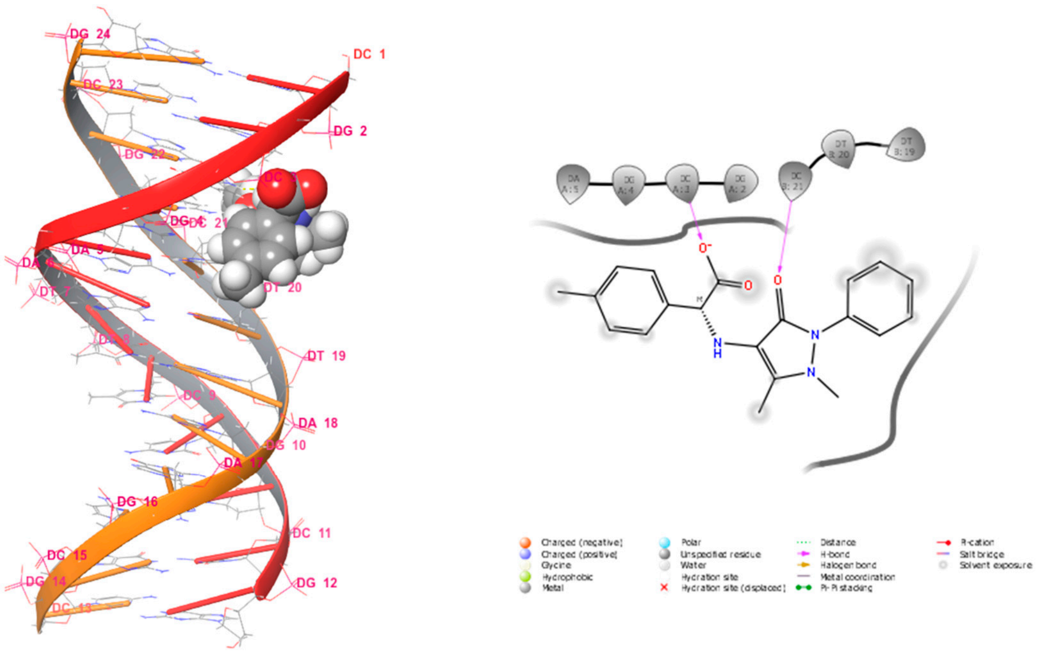

Figure S27: The 3D binding model of **8** with B-DNA dodecamer (1BNA) (**A**), 2D diagram of **8** and interacting residues (**B**)

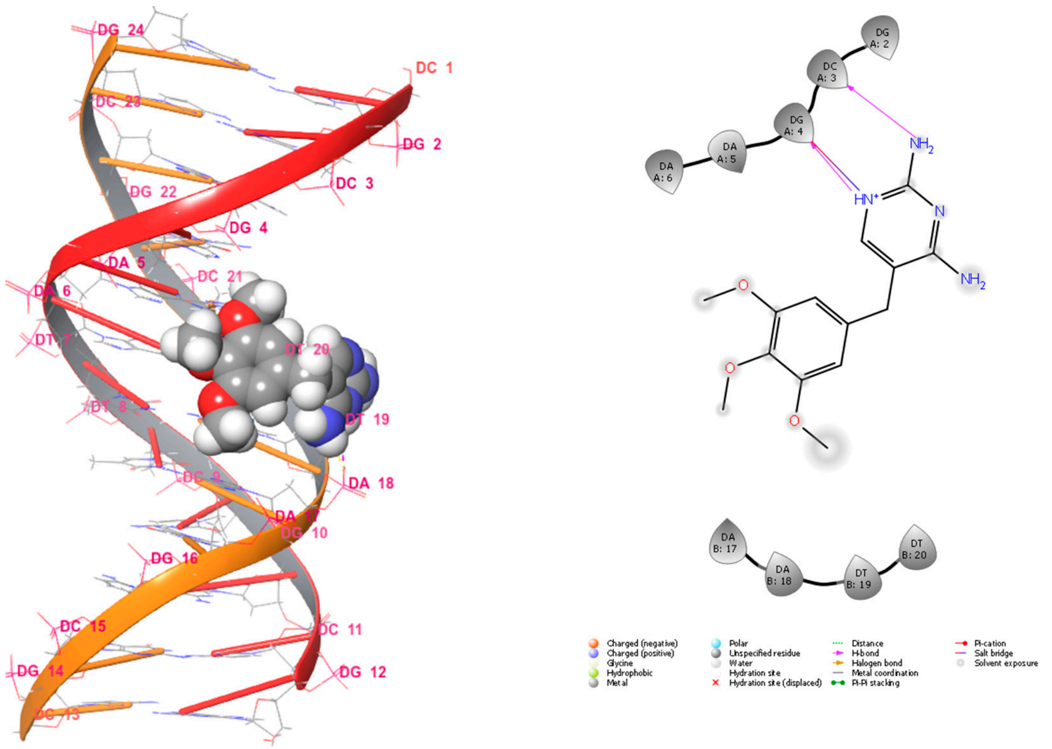

Figure S28: The 3D binding model of **Trimethoprim** with B-DNA dodecamer (1BNA) (**A**), 2D diagram of **Trimethoprim** and interacting residues (**B)**

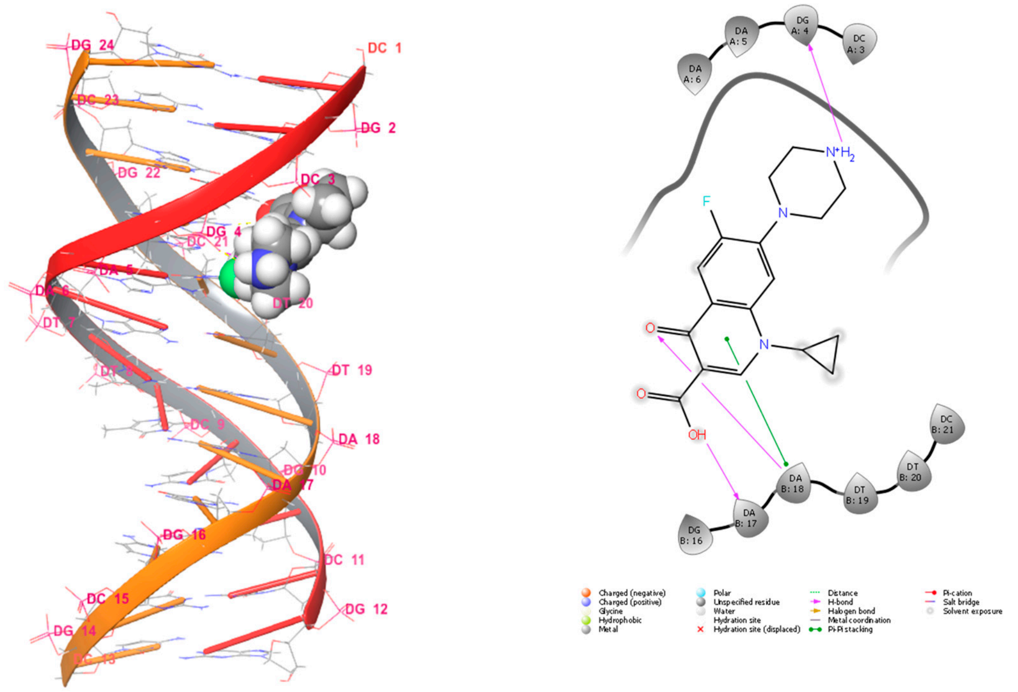

Figure S29: The 3D binding model of **Ciprofloxacin** with B-DNA dodecamer (1BNA) (**A**), 2D diagram of **Ciprofloxacin** and interacting residues (**B**)

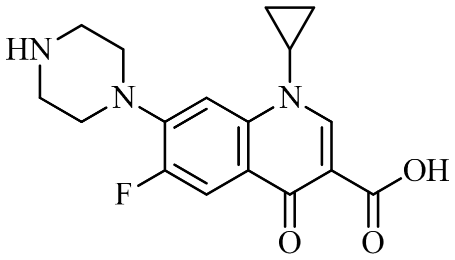

Ciprofloxacin

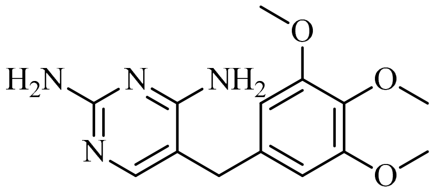

Trimethoprim

Figure S30: Structures of standard drugs

Table S1: Binding free energy components for the B-DNA dodecamer (1BNA)-ligand complexes calculated by MM-GBSA

**Ligands**	**B-DNA DODECAMER (1BNA)** (kcal/mol)							
	**ΔGbind**	ΔG_{Coul}	ΔG_{cov}	ΔG_{Hbond}	ΔG_{Pack}	ΔG_{lipho}	ΔG Solv_GB	ΔG vdW
Schiff Bases								
**1**	-18.56	-5.11	2.16	0.00	0.00	-6.25	16.22	-25.57
**2**	-35.43	-456.96	5.65	0.00	0.00	-8.24	439.88	-15.76
**3**	-22.71	-24.63	-0.07	0.00	0.00	-6.63	27.10	-18.48
**4**	-17.20	-392.40	5.23	0.00	0.00	-7.09	404.077	-27.01
**α-unsaturated aminocarboxylic acids**								
**5**	-6.39	260.30	0.90	0.00	0.00	-5.32	-237.31	-24.97
6	-14.03	69.78	5.01	0.00	0.00	-5.55	-64.21	-19.06
7	-1.07	258.78	3.85	0.00	0.00	-6.97	-230.60	-26.12
**8**	0.23	258.32	8.38	0.00	0.00	-7.73	-232.11	-27.09
**Control**								
**Trimethoprim**	-29.52	-533.48	1.36	0.00	0.00	-5.40	533.25	-25.25
**Ciprofloxacin**	-36.19	-205.91	2.58	0.00	0.00	-6.48	197.18	-22.34

ΔG Bind: Binding free Energy; ΔG Coul: Coulomb or Electrostatics Interaction energy; ΔG_{cov}: Covalent bonding correction, ΔG_{Hbond}: Hydrogen-bonding correction, ΔG_{Pack}: π-π packing correction, ΔG_{lipho} Lipophilic Interaction energy, ΔG Solv_GB: Generalized Born electrostatic solvation energy, ΔG vdW: Van der Waals Interaction energy.

ADMET properties

Table S2: Range and recommended value(s) of ADMET properties

**Property**	**Description**	**Range or recommended value (s)**
Mw	Molecular weight (MW in g/mol)	130.0 – 725.0
#Stars	Number of property or descriptor values that fall outside the 95% range of similar values for known drugs.	0 – 5
WPSA	Weakly polar component of the SASA (halogens, P, and S).	0.0 – 175.0
volume (A^3)	Total solvent-accessible volume in cubic angstroms using a probe with a 1.4 Å radius.	500.0 – 2000.0
QPpolrz (A^3)	Predicted polarizability in cubic angstroms	13.0 – 70.0
EA (eV)	Calculated electron affinity	–0.9 – 1.7
QPlogPoct	Predicted octanol/gas partition coefficient.	8.0– 35.0
QPlogPw	Predicted water/gas partition coefficient.	4.0 – 45.0
QPlogPo/w	Predicted octanol/water partition coefficient.	–2.0 – 6.5
QPlogS	Predicted aqueous solubility, log S. S in mol dm–3 is the concentration of the solute in a saturated solution that is in equilibrium with the crystalline solid.	-6.5 – 0.5
QPPCaco (nm/sec)	Predicted apparent Caco-2 cell permeability in nm/sec. Caco-2 cells are a model for the gut-blood barrier.	<25 poor,<br>>500 great
#metab	Number of likely metabolic reactions	1 – 8
%Human Oral Absor	Predicted human oral absorption on 0 to 100% scale	>80% is high<br><25% is poor
PSA	Van der Waals surface area of polar nitrogen and oxygen atoms.	7.0 – 200.0

Rule of 3	Number of violations of Jorgensen's rule of three.	0
Rule Of Five	Number of violations of Lipinski's rule of five	4

Reference

[1] R. Iakovenko, J. Hlaváč, Visible light-mediated metal-free double bond deuteration of substituted phenylalkenes, Green Chemistry, 23 (2021) 440-446.
